# Small-Molecule Trk Agonists: Where Do We Go from Here?

**DOI:** 10.1021/acs.jmedchem.4c02365

**Published:** 2025-07-18

**Authors:** Tye S. Thompson, Arthur Sefiani, Kevin Burgess

**Affiliations:** † Department of Chemistry, Texas A&M University, Box 30012, College Station, Texas 77842-3012, United States; ‡ Department of Neuroscience and Experimental Therapeutics, 14736 Texas A&M University Health Science Center, Bryan, Texas 77807-1552, United States

## Abstract

About two decades ago, there were no validated, agonistic small-molecule modulators of Trk (tropomyosin receptor kinase). High-throughput screening of commercial libraries seemed an attractive way to identify starting structures when the research community became aware of their potential for treatment of neurodegeneration and traumatic injuries (e.g., stroke) because this strategy avoids high-level chemical expertise for molecular design and synthesis. Cost and effort constraints arising from library acquisition and assays imposed limitations on numbers of compounds tested, so filtering to reduce library sizes before screening was routine. One of the criteria was to prioritize existing pharmaceuticals because these had known toxicity profiles and side effects, at least in some delivery and dosing regimens, and many cases had proven blood brain barrier (BBB) permeabilities. This review gives our perspective on how these efforts transpired, lessons learned, and constraints which hold back development in this area at the present time.

## Graphical abstract

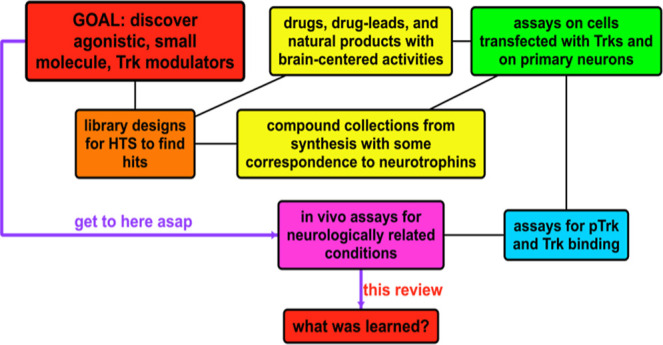

## 1 Introduction

### 1.1 Development of Small-Molecule Probes

Small-molecule probes are widely used to elucidate cell signaling effects and as foundations for therapeutic studies.^1^ An ideal strategy for probe development might feature libraries of readily accessible candidate compounds, a convenient primary assay, secondary assays, and then in vivo studies to select leads from hits. There are numerous instances of this, and their success in probe development varies nonuniformly with target receptors because the challenges vary too.^2^

Probe development for cell surface receptors can encompass several challenges.^3^ First, structural data to guide probe design can be limited by difficulties crystallizing transmembrane receptors in environments that closely resemble native states found in membranes. Second, many interesting cell surface receptors modulate cell signaling pathways via protein–protein interactions (PPIs).^3^ It tends to be difficult to design small molecules that modulate PPIs relative to other receptor types.^4^ These problems are accentuated when receptor activities are dictated by protein oligomers on the surface, which may not be recapitulated in crystallization for X-ray studies or in biochemical assays. Third, secondary assays are relatively limited for monitoring some biological outcomes. For instance, compound potency for many cancer-related targets can be accessed via cytotoxicity featuring select tumorigenic cells, which tend to be easy to obtain and culture, followed by relatively convenient in vivo assays to assess tumor size.^5^ However, it is harder to construct than to destroy. In vivo assays featuring otherwise healthy but aging or damaged tissue types, for instance, in models for neurodegeneration or prevention of neurodegeneration tend to be more difficult.

Library design criteria are especially important if the easier preliminary assays are not definitive, and later in vivo assays are time-consuming, involve expensive models, or require high level expertise.^6^ Under these circumstances, it is difficult to prioritize tentative hits for selection of robust leads, so then small libraries based on good design criteria are much preferred to high-throughput screening of largely random compound collections. However, if structural data are limited, what criteria can be used to guide the compound design to construct those focused libraries?

### 1.2 NT·Trk·p75 Interactions

Neurotrophins (primarily nerve growth factor (NGF); brain derived neurotrophic factor, (BDNF); neurotrophin-3 (NT-3); and neurotrophin-4, (NT-4)) are cytokines that interact selectively, but not specifically,^7^ with a series of related tropomyosin receptor kinases (TrkA·NGF; TrkB·BDNF; TrkB·NT-4; and TrkC·NT-3). Figure 1a illustrates these primary interactions and their cross-reactivities. For instance, NT-3 binds TrkA but with lower affinity than for C.^8,9^ All of the neurotrophins (NTs) interact with another receptor, p75. p75 has been called the “death receptor”^10^ because it induces apoptosis, but this is an oversimplification because it also works in synergy with Trks to modulate their activity and sometimes to promote survival.^11–17^ For instance, expression of p75 can determine whether NT-3 binds and activates TrkA.^18–20^

**Figure 1:**
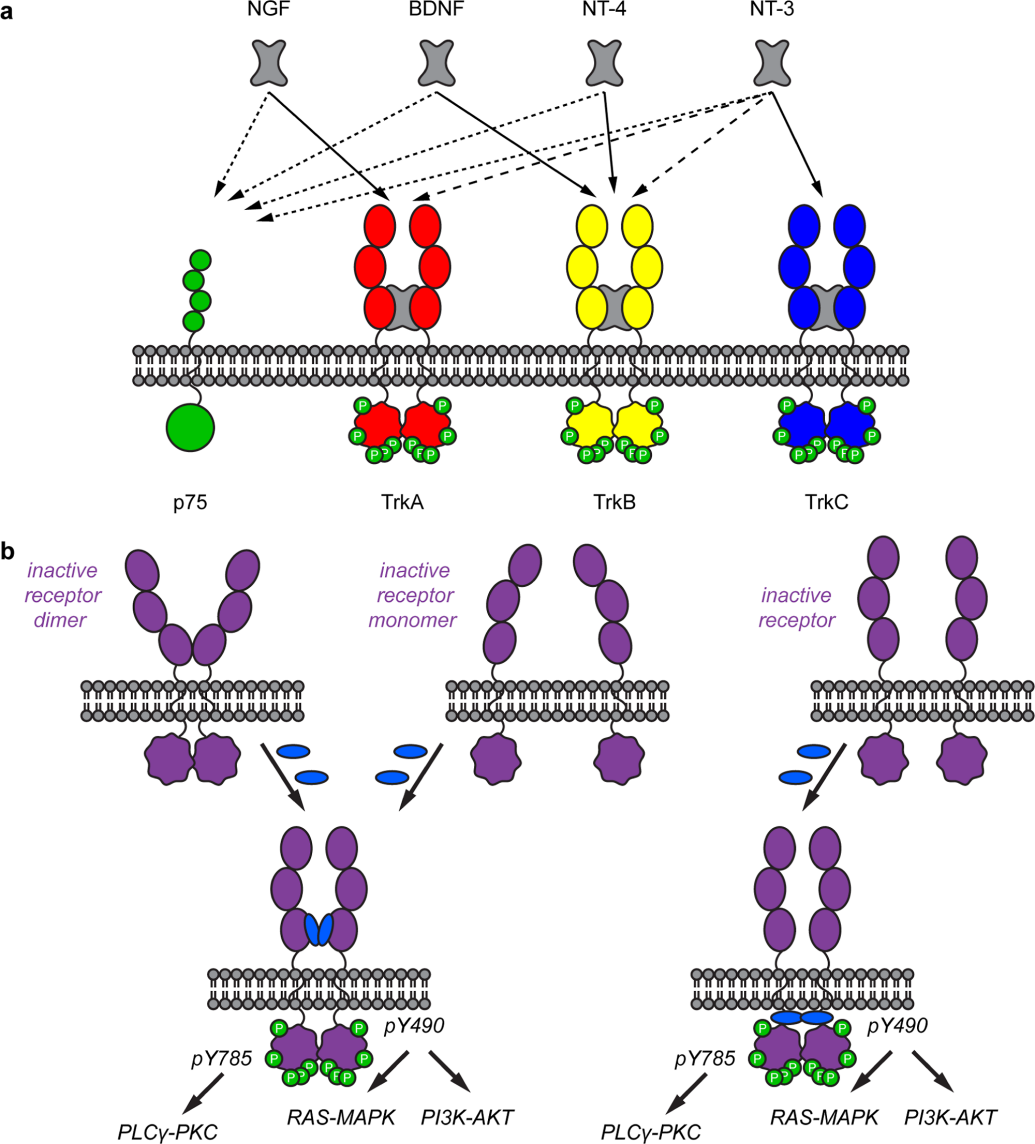
(a) Neurotrophins are dimeric (gray) and interact selectively with their primary targets (black arrows) but also show cross-reactivity with other Trks (broad dashed line). All the Neurotrophins interact with p75 (thin dashed line). (b) Some mechanisms by which monomeric small molecules might activate Trk receptors.

Trk receptors have evolved to induce related but different effects depending on the signaling pathways they activate. Generally, pathways activated by different neurotrophins and Trk receptors can determine neuronal growth and differentiation outcomes.^21–24^

Neurotrophins exist as disulfide-linked dimers in solution, and they do not change on binding Trks.^25^ However, structural changes in the Trk receptors lead to autophosphorylation of intracellular tyrosine (Tyr) residue combinations in and around their Tyr kinase domains. Subsequently, signaling pathways are stimulated, leading to protein expression and cellular transformations. NT interactions with all Trks tend to stimulate at least PI3K-AKT, RAS-MAPK, and PLCγ-PKC signaling, but other pathways may be involved too.

Evidence from production of mutated and chimeric NTs suggests hot segments^4^ in these dimers are localized in hot loops^26–29^ and on short *N*-terminal sequences.^30^ In this article, the three loops are numbered 1–3 (the order in which they occur in the NT sequences *N*-to-*C*) and are color coded red (1), green (2), and blue (3). In Figure 2, the *N*-terminal fragment is specifically labeled: this interacts with the Trks. These regions are highly variable between NTs and control, at least their Trk selectivities (“hot” implies small peptide regions in the protein ligands, here NTs, especially important for binding their receptors, e.g., Trks). Crystal structures of NTs presented as ribbon or tube representations are almost indistinguishable because they comprise homologous cysteine-knotted β-sheet regions (gray),^31^ providing a similar structural core to present these loop and helical selectivity determinants.

**Figure 2:**
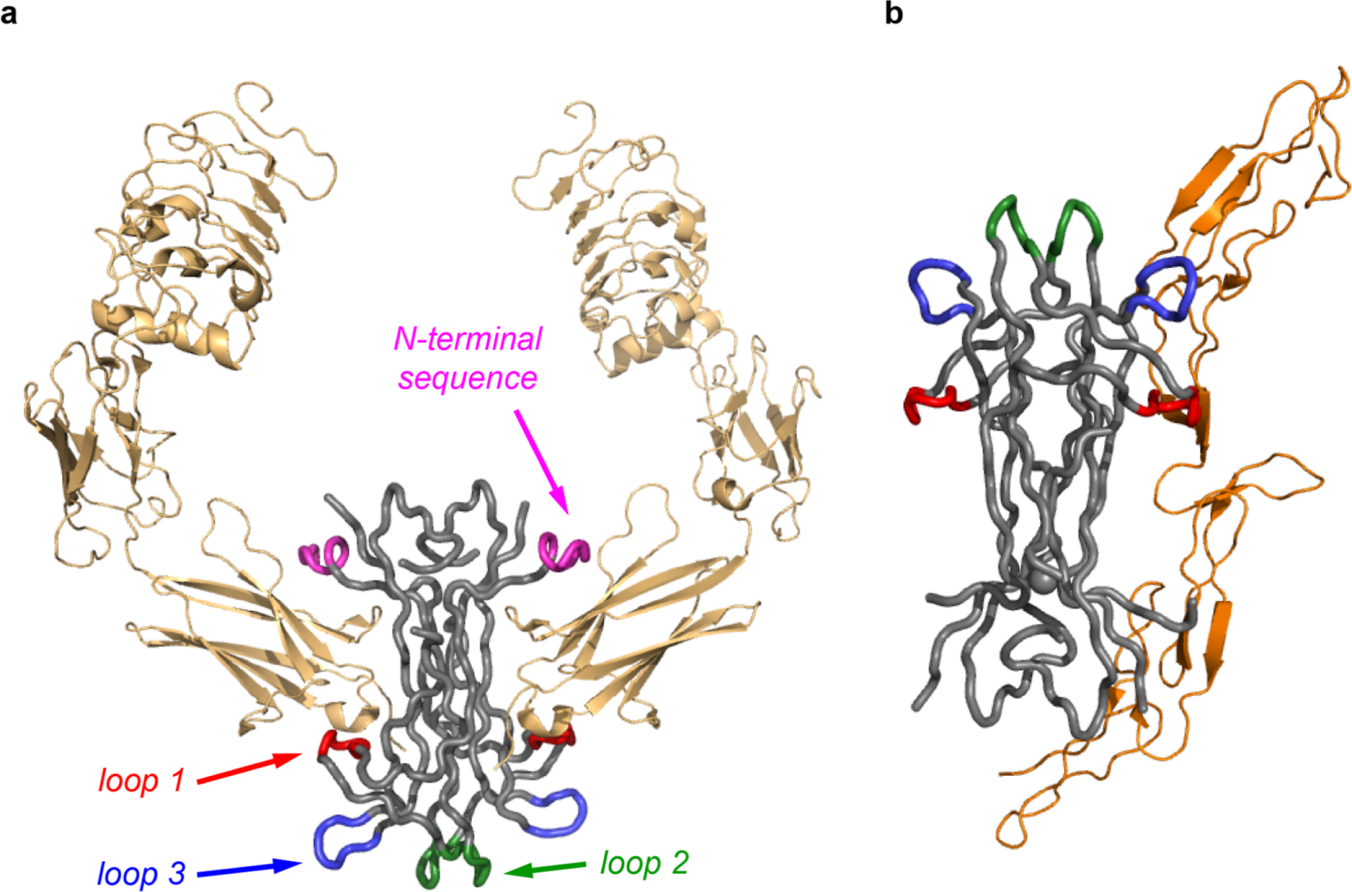
(a) NGF interaction with the extracellular domain of TrkA (PDB 2IFG). (b) NGF interacting with the extracellular domain of p75 (PDB 1SG1).

Computer-aided molecular design of small molecules to mimic these selectivity determinants requires structural data, but this is one of the problems alluded to in the first section. The structure in Figure 2 is typical of all NT·Trk structural data insofar as only a Trk *fragment* (the extracellular domain of TrkA) was crystallized. This partial structure represents much surface area contact between NGF and TrkA, but unfortunately, contacts between two of the highly variable selectivity determining regions of NGF (loops 2 and 3) and the linker region they interact with remain unresolved.

Crystallographic analyses of NT·Trk interactions are limited to extracellular domains, as illustrated in Figure 2, because crystallization of complete proteins would require a hydrophilic medium for the extracellular domain and a hydrophobic one for the transmembrane region; this is hard to arrange. It is also difficult to express complete Trks for similar reasons, and the proteins are vulnerable to proteolytic degradation throughout. Finally, it is impossible to be sure Trk fragments successfully cocrystallized with NTs represent the structure in natural cell environments.

Analogies of Trks with other receptor tyrosine kinases imply the extracellular domains are joined by a linker region to transmembrane helices and then to the intracellular parts. Interaction of NT loops with that linker region is critical for NT binding selectivity. For example, expression of TrkB with a truncated linker region gave neurons that still bound BDNF, but not NT-4 or NT-3.^32,33^ These truncated isoforms are differentially expressed in different neuron populations, which could be one mechanism through which responsiveness to select neurotrophins over others is modulated.

### 1.3 Do Small-Molecule Trk Ligands Have to Be Dimeric to Induce Signaling?

For cell surface receptors in general, activation by *monovalent* ligands *has* been inferred, though not necessarily widely validated by other researchers, and several mechanisms have been suggested (Figure 1b), some of which are discussed extensively later in this review (see particularly the section 6). Thus, receptors may be naturally oligomerized in membranes and then activated by monovalent ligands (1b, left).^34^ For instance, the leptin receptor (OB-R) exists as preformed, inactive, dimers that are conformationally perturbed by leptin giving downstream signaling.^35^ The erythropoietin receptor (EPOR) similarly exists as preformed dimers^36^ that conformationally reshuffle on EPO binding to give intracellular signaling.^37^ Epidermal growth factor (EGF) is unlike the NTs insofar as two EGF molecules bind *outside* EGFR dimers giving receptor activation;^38^ furthermore, binding triggers a conformational shift in the extracellular region, allowing the monomeric receptor to dimerize and activate (1b, center). The fibroblast growth factor FGFR and insulin receptors function through similar mechanisms.^39^ Some monovalent ligands could bind allosteric sites distal to where the native ligand docks yet still induce activation (1b, bottom right).^40,41^

A P203A mutation in the TrkA extracellular domain induces spontaneous receptor dimerization and activation independent of any NGF or other TrkA ligands.^42^ The inference is any force inducing an appropriate Trk conformational change in the extracellular region can induce receptor dimerization and activation. Hence, the first two potential mechanisms of monovalent molecule activation could occur through binding to either an allosteric site or the neurotrophin binding region itself.

It transpires some small molecules can induce effects which resemble Trk activation without directly agonizing the receptor. In this article, we favor the term “Trk modulators” in cases where there is mechanistic ambiguity, but the effects are occurring through Trks. Other small molecules may perturb NT·p75 interactions.^43^ In cases like this, even the expected outcome is unclear. Agonist may induce cell death or apoptosis, but antagonists could induce trophic effects characteristic of direct Trk agonism.

There are many other possibilities for which small molecules could induce neurotrophic effects without directly perturbing Trk tertiary structures. They could up- or down-regulate NT or Trk expression levels, activate other proteins that result in observed neurotrophic effects, or activate several receptors, including Trks, causing cell survival and/or proliferation via polypharmacology. It can be difficult to prove that these pathways are not involved for putative Trk agonists and modulators.

### 1.4 Scope of This Review

Trk inhibitors, particularly of Trk receptor variants, are interesting for oncology,^44,45^ but they are not part of this review on agonists. This review also deliberately excludes peptide and peptidomimetics with Trk agonists; we plan to deal with those elsewhere. Peptide/peptidomimetics have been covered before from different perspectives. For instance, a review on low-molecular-weight mimetics of NGF and BDNF provides an overview of both small molecules and peptide/peptidomimetics but with a focus on dimeric dipeptide mimics.^46^ Two recent reviews discuss TrkB, BDNF, and BDNF mimics in Alzheimer’s disease^47,48^ and another their impacts on central nervous system injury.^49^ A broader range of specifically peptide mimetics of NGF, BDNF, glial cell-line-derived neurotrophic facto (GDNF), HGF, PDGF, and FGF as potential treatments for traumatic brain injury was discussed in another recent review.^50^ Finally, one recent review asks if any kind of small molecules are even the right tool to explore the therapeutic potential BDNF, highlighting the potential of Abs as TrkB agonists, which they suggest are superior to small molecule agonists.^51^

## 2 Small-Molecule Trk Modulators

### 2.1 From High-Throughput Screening of Biologically Relevant Libraries

The Ye group at Emory extensively used this strategy to discover TrkA and TrkB small-molecule agonists, and their work is often discussed here. Screens of biologically relevant small molecules (∼2000 compounds from the Spectrum Collection Library) for Trk agonists have generated several hits. The screening strategy typically was for the rescue of murine basal forebrain SN56 cells stably transfected with TrkA or B from apoptosis (against a SN56 TrkA- and B-counter screen) and then qualitative neurite outgrowth assays. Selected hits were analyzed for Trk activation and downstream signaling via blotting, as well as several other in vitro and in vivo secondary assays. Compounds found to exhibit neurotrophic activity in this manner include gambogic amide^52^ (TrkA agonist), amitriptyline^53^ (A and B agonist), and the B agonists *N*-acetyl serotonin^54^ (NAS), deoxygedunin,^55^ and 7,8-dihydroxyflavone (7,8-DHF).^56^ Small-molecule Trk modulators discovered by other groups include DMAQ-B1 and deprenyl.
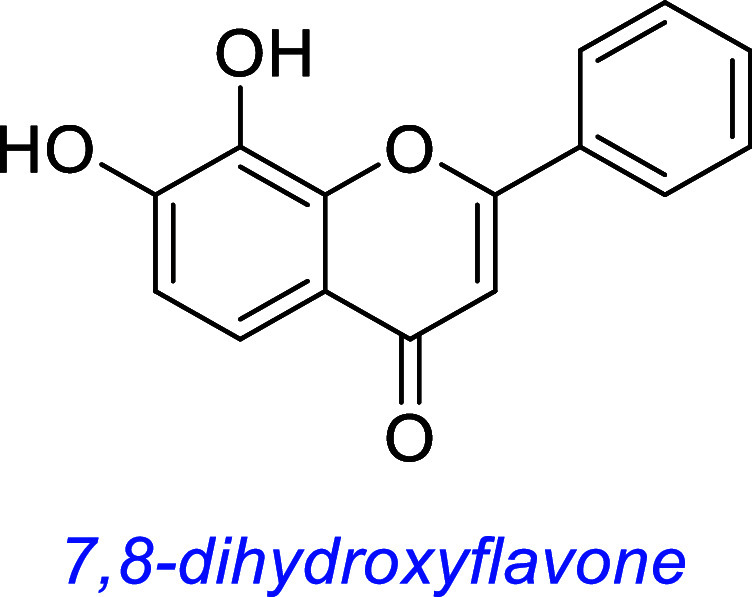


*7,8-Dihydroxyflavone* (7,8-DHF) also was identified in screens by Ye and co-workers featuring TrkB-expressing transfectants.^56^ Like many other flavones or flavonoids, it occurs naturally in leaves of certain tree species.^57^ This review describes studies in which agonism or modulation by 7,8-DHF has been contested by at least five laboratories. On the other hand, many in vivo studies, some from Ye and many from other groups, indicate this compound gives outcomes expected to be, or associated with, signaling through TrkB.

In the original screens,^56^ five flavones were identified in apoptosis assays of SN56 and T48 cells, as previously discussed, of which 7,8-DHF was most active. Several other flavones were inactive in the same assay. Immunofluorescence staining with antiphospho-TrkB-Y816 indicated 7,8-DHF activates TrkB in hippocampal neurons and phosphorylation of TrkB, AKT, and ERK (immunoblotting and inhibited by cotreatment with K252a). 7,8-DHF was also observed to activate TrkB in vivo in the cortex of wild type and conditional BDNF knockout mice. It was found to induce TrkB dimerization as potently as BDNF (but did not induce TrkA dimerization) and, in radiolabeled and GST pulldown experiments, bound to the TrkB extra-, but not the intra-, cellular domain, TrkB. It did not bind the intracellular domain of TrkA. 7,8-DHF also prevented kainic-acid-induced neuronal apoptosis in the mouse hippocampus in a TrkB-dependent manner (not effective in induced TrkB-null knockin mice), reduced infarct volumes in a stroke model, and prevented induced neurotoxicity in a mouse model of Parkinson’s disease. Finally, 7,8-DHF was shown to suppress TrkB-dependent glutamate-induced caspase-3 activation in mouse primary cortical neurons.

Recent reviews of NGF and BDNF mimetics discuss many 7,8-DHF studies in more detail,^46,49,51,58^ and its activity as a TrkB agonist is seemingly well-characterized. On the other hand, therapeutic effects of 7,8-DHF are questionable. In an Alzheimer’s disease (AD) mouse model, 7,8-DHF did not treat amyloid pathology or improve cognitive function.^51^ At 10 μM, 7,8-DHF caused detrimental effects on porcine oocytes and embryos, including increased intracellular reactive oxygen species (ROS) and lower developmental competence of porcine parthenogenetic embryos.^59^ Adverse effects of 7,8-DHF can be sex-dependent. In female mice, 7,8-DHF may mitigate adiposity and weight gain, but it can worsen adiposity and adipose inflammation in male mice.^60^ There is limited clinical data, but 7,8-DHF-induced nausea, dizziness, overstimulation, irritability, and insomnia have been reported.^61^ 
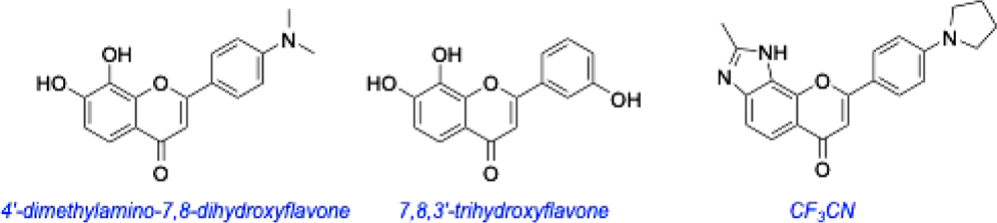


A series of 7,8-DHF derivatives have also been screened. These have been less well-studied, but both 7,8,3′-trihydroxyflavone (7,8,3′-THF) and 4′-dimethylamino-7,8-dihydroxyl flavone (4′-DMA-7,8-DHF) were demonstrated to have higher antiapoptotic activity and TrkB phosphorylation than 7,8-DHF in vitro, and 4′-DMA-7,8-DHF demonstrated potent antidepressant activity in a forced swim test.^62^ Another analogue, CF_3_CN, has been reported^63^ to interact with the leucine rich motif and cysteine cluster 2 domain of the TrkB extracellular region (overall *K*_d_ 80 nM). It activates TrkB neurotrophic signaling in primary neurons and mouse brains. Oral administration of CF3CN blocks delta-secretase activation, attenuates AD pathologies, and alleviates cognitive dysfunction in 5xFAD mice. These are transgenic mice rapidly that can display features of Alzheimer’s disease amyloid pathology, intraneuronal Aβ-42-induced neurodegeneration, and amyloid plaque formation.
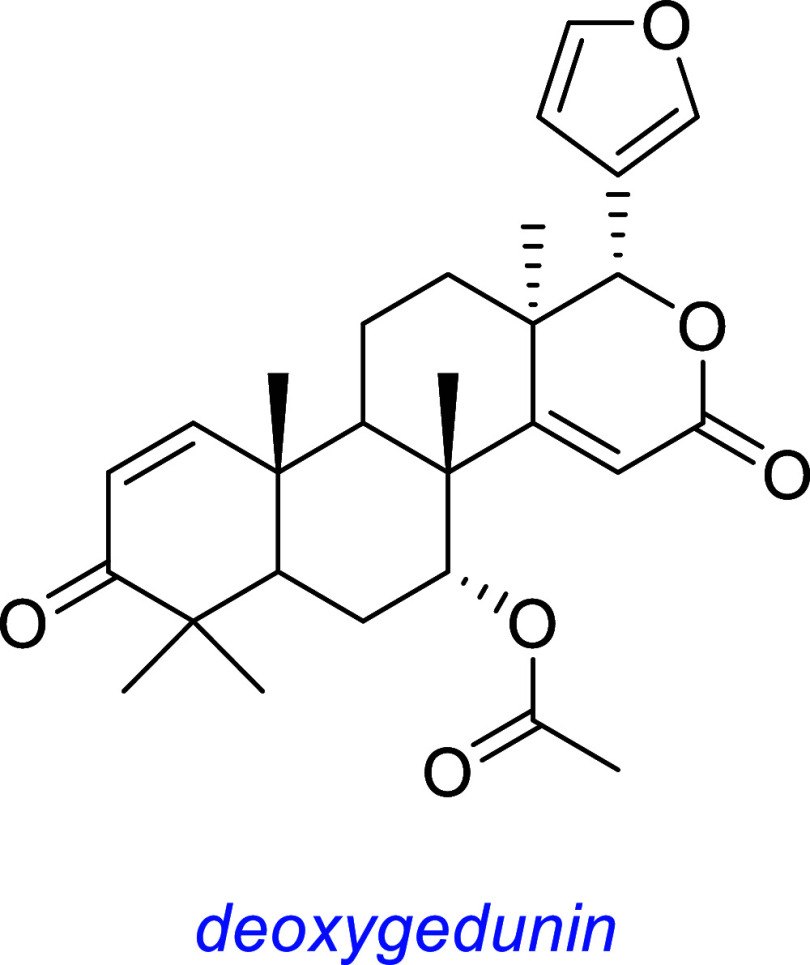


*Deoxygedunin*, a natural product occurring in the neem tree, was identified as a hit from HTS via an apoptosis assay in SN56 basal forebrain cells transfected with TrkB (Ye group^55^). It was reported to increase survival of primary hippocampal neurons that express TrkB and bind to the TrkB extracellular domain, triggering dimerization, phosphorylation, and activation of AKT and ERK. In vivo, it activated TrkB in wild type mice and in TrkBF616A knock-in mice (resulting in a TrkB-null phenotype upon treatment with 1NMPP1, a TrkBF616A inhibitor), indicating a BDNF-independent and TrkB-dependent mechanism of action. However, downstream effects of deoxygedunin seem to be like those of BDNF because it exhibits antidepressant effects and enhances fear acquisition in vivo. Ye and co-workers reported deoxygedunin promotes axon regeneration in cut peripheral nerves in mice^64^ but not in selective TrkB-knock out mice, strongly implicating a TrkB-dependent mechanism. It was also found to induce TrkB/AKT/MAPK-dependent neuroprotective properties in mouse and rat models of Parkinson’s disease.^65^ 
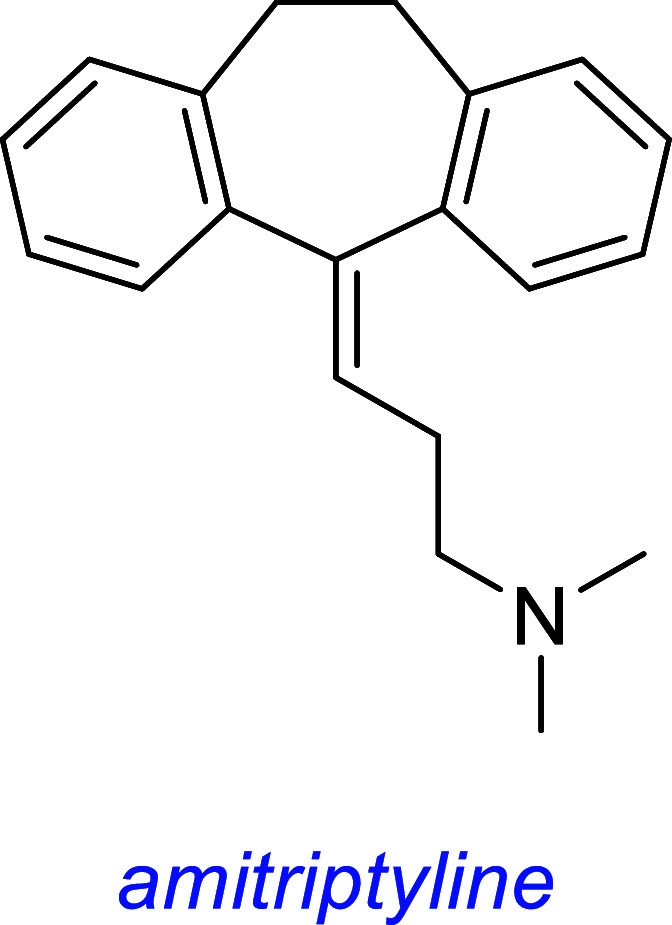


*Amitriptyline* (Elavil) is a second line antidepressant used to treat major depressive disorder, neuropathic pain, fibromyalgia, migraine, and tension headaches.^66^ It is not a preferred therapy because of frequent and prominent side effects. Further, it interacts with a variety of receptors (e.g., some for serotonin, histamine, and acetylcholine). It is hydrophobic, and this is often associated with nonspecific binding in vivo, consistent with its known polypharmacology.

It was highlighted in the same cell survival screen as that of gambogic amide when it protected TrkA-transfected SN56 cells from apoptosis.^53^ Subsequently, it was found to protect primary hippocampal neurons from glutamate-induced apoptosis in vitro and induce TrkA Y751 (kinase activation loop and PLCγ recognition site) phosphorylation but not at Y490 (SHC recognition site). Amitriptyline-induced phosphorylation of TrkA was inhibited by the Trk kinase inhibitor K252a. Amitriptyline also induced transient (10–30 min) pAKT and sustained (30 to >180 min) pERK in primary hippocampal neurons.

Amitriptyline binds the extracellular domain of TrkA and B with *K*_d_ values of 3 and 14 μM, respectively (assayed against ^3^H-labeled amitriptyline), triggers Trk dimerization and autophosphorylation, and induces neurite outgrowth of PC12 cells. This compound also robustly activates TrkA and TrkB in vivo, prevents kainic-acid-induced neuronal death, and triggers formation of TrkA/B heterodimers.

Another group performed NMR experiments to elucidate TrkA-bound conformation(s) of amitriptyline: it showed an overlap with the NGF binding region and displaced ^125^I-labeled NGF on full-length TrkA expressed in HEK293 cells.^67^ A third group found amitriptyline protected against hypoxia–reoxygenation-induced apoptosis of primary mouse cardiomyocytes in vitro through a TrkA-AKT-dependent mechanism.^68^

Ye^53^ initially reported TrkA and B activities, but two groups^69,70^ reported unsuccessful attempts to reproduce agonism or modulation of TrkB. Their contrary findings are summarized in this review after those other molecules are discussed.

Several serious side effects are associated with amitriptyline. It causes significant damage to the liver and kidney,^71^ aberrant neurite outgrowth patterns and death at clinically relevant concentrations,^72^ and “Wallerian” anterograde degeneration of distal ends of axons in peripheral nerve fibers in vivo.^73^ For these reasons, its clinical use is controversial.
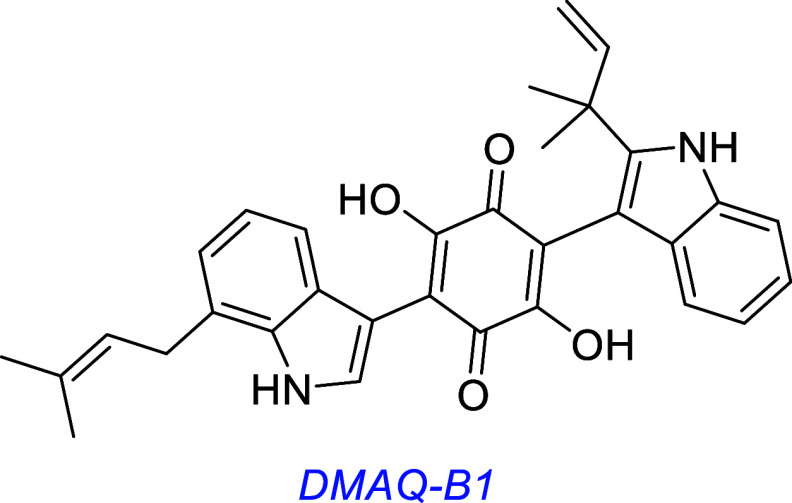


*Demethylasterriquinone-B1 (DMAQ-B1, also called DAQ-B1, or L-783,281)* is a fungal metabolite identified as an insulin receptor (IR) agonist^74^ screening for tyrosine kinase activity of the immunopurified IR receptor. Conversely, it did not activate IGF1R, EGFR, and PDGFR. DMAQ-B1 induces phosphorylation of the insulin receptor, AKT and PI3K, and exhibited antidiabetic activity in mice. Most studies of DMAQ-B1 explore its IR agonistic activity; Trks have been relatively less well-studied.

A different group investigating potential RTK (receptor tyrosine kinase) targets of DMAQ-B1 found it activates Trk receptors to varying degrees.^75^ DMAQ-B1 was also found to activate Trk in rat and human primary cortical neuron cultures and in rat DRG neuron cultures. It appears to target intracellular Trk domains and induce receptor dimerization based on the absence of phosphorylation for Trk·PDGFR extracellular–intracellular chimeric constructs, but pTrk was observed for PDGFR·Trk constructs; intact PDGFR is *not* activated by DMAQ-B1.

TrkA-, B-, and C-transfected CHO cells treated with DMAQ-B1 were lysed, Trk was immunoprecipitated, and then, pTyr was assessed via immunoblotting with an anti-pTyr antibody (Ab, 4G10). DMAQ-B1 stimulated pTrkA up to 41% of the maximal levels induced by NGF and pTrkB and C phosphorylation by 37 and 14% of those induced by BDNF and NT-3, respectively. In the same assay, DMAQ-B1 showed maximal efficacy at the shortest time point tested (3 min) with decreasing but detectable activity until 180 min. In contrast, the level of NGF-induced pTrkA remained high over this time. Initially, DMAQ-B1 was found to robustly activate pAKT and pERK in TrkA- and B-expressing cell lines. However, interpretation of these data is complicated by the insulin receptor in CHO cells. Thus, insulin also evoked pAKT in the transfectants, and pERK and pAKT were observed for DMAQ-B1-treated, untransfected CHO cells. Further, DMAQ-B1 induces pPLCγ in TrkA-expressing CHO cells but also in wild-type cells, though substantially less.

DMAQ-B1 was tested in a cell survival experiment in primary rat cortical neurons, but it was cytotoxic above 10 μM, and this concentration was necessary to observe pTrk. This type of cytotoxicity likely limits studies of DMAQ-B1 in vivo.

Low levels of TrkB phosphorylation were observed via a sandwich ELISA in HEK293-TrkB transfectants, though not in primary cortical neuron cultures.^70^ ERK and AKT activation was also observed in the same study via an enzyme-linked fixed-cell immunoassay in the primary cortical neuron cultures.^70^ 
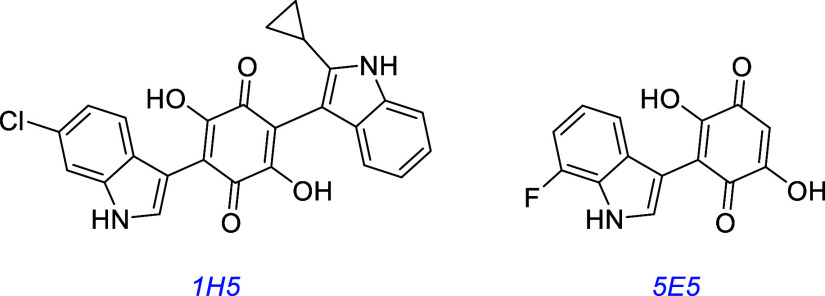


A library of synthetic asterriquinones and monoquinones with structures like DMAQ-B1 has been prepared and tested. Two less toxic derivatives (1H5 and 5E5) were found with equivalent or greater biological efficacy in receptor activation, cell survival, and neurite outgrowth experiments in Trk transfectants and PC12 cells.^76^ 
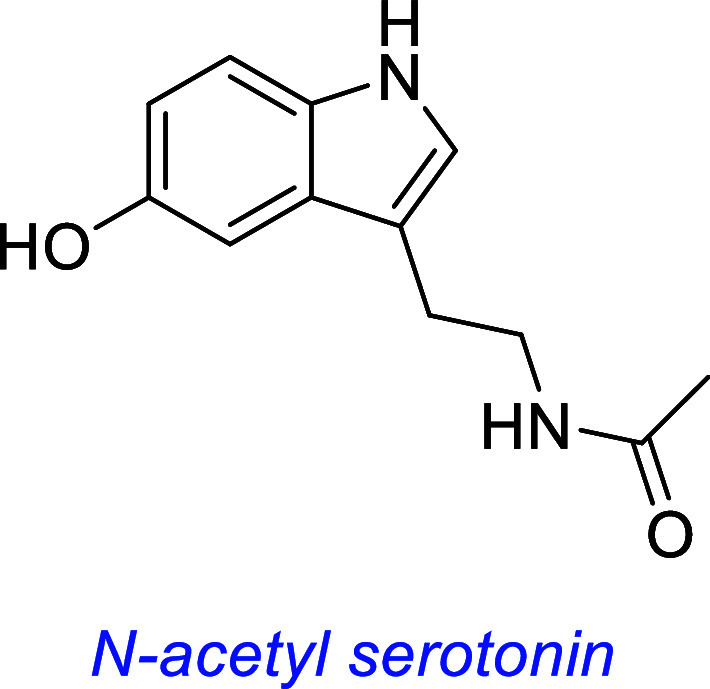


*N-Acetyl Serotonin* (NAS) occurs naturally as an intermediate in the production of melatonin from serotonin. It was reported to trigger phosphorylation of TrkB, AKT, and ERK in primary hippocampal neurons.^54^ Induced phosphorylation was diminished with concurrent treatment of neurons with NAS (or BDNF control) and kinase inhibitor K252a, indicative of a TrkB-dependent mechanism. It stimulated pTrkB production in primary cortical neurons and in vivo in retina and hippocampus tissue. However, another group has disputed that NAS is a TrkB agonist or modulator (see below).^69^ 
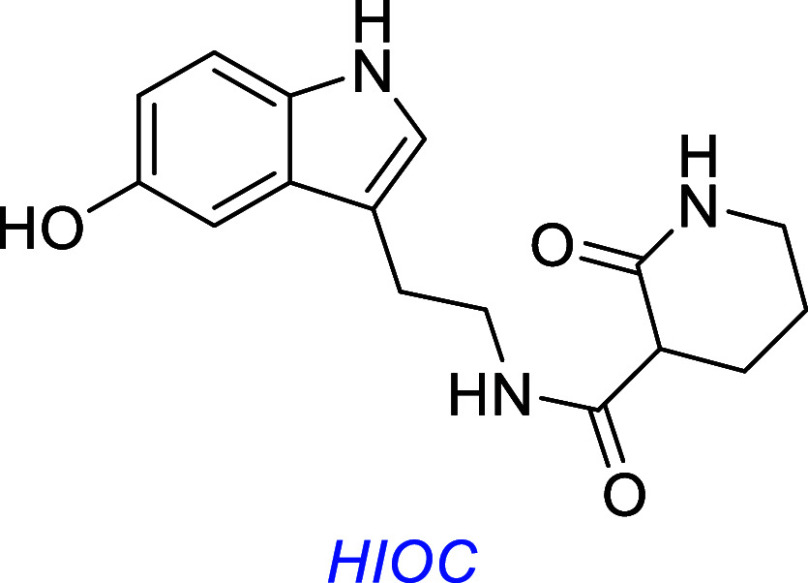


The NAS derivative HIOC (*N*-{2-(5-hydroxy-1*H*-indol-3-yl)­ethyl}-2-oxopiperidine-3- carboxamide) has been reported to putatively activate TrkB and protect against light-induced retinal neurodegeneration in vivo,^77^ and another group subsequently found it protects against induced vision loss in a mouse model.^78^ HIOC has yet to be evaluated for safety concerns commonly associated with putative TrkB agonists.
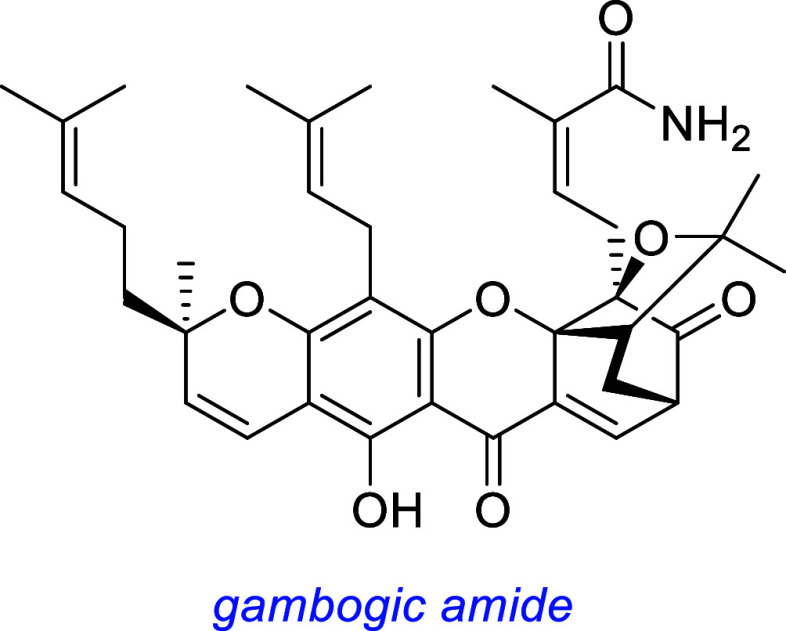


*Gambogic amide* was reported to prevent apoptosis in TrkA-transfected SN56 cells but not in SN56 cells.^52^ Blotting indicated it triggers pTrkA (at Y490 and at Y751 based on Ab selectivities), pAKT, and pERK. Experiments with GFP-tagged, truncated TrkA receptors expressed in HEK293 cells showed gambogic amide associated with the juxtamembrane domain (intracellular region connecting the transmembrane domain to the tyrosine kinase domain), permeating into these TrkA-expressing cells. Affinity experiments using gambogic amide as bait captured the juxtamembrane regions of wild-type TrkA and less of kinase-dead TrkA, but it did not bind TrkB or TrkC. The same beads were used to compete free gambogic amide with that on the affinity bead, for GFP-TrkA; hence, a *K*_d_ of ∼ 75 nM was estimated. FITC-conjugated gambogic acid penetrated PC12 cells (embryonic rat pheochromocytoma) and associated with TrkA, but not when the SHC binding region of TrkA was truncated.

Gambogic amide was shown to protect mouse hippocampal neurons in vivo from kainic acid-induced neurodegeneration and reduce infarct volume in vivo in rats following a middle cerebral artery occlusion (MCAO) stroke model (2 mg/kg, single dose before restoration of blood flow).

Another group subsequently found gambogic amide upregulates TrkA expression both in vitro and in vivo and activates TrkA in mouse hippocampus and suggested this could be the mechanism of its neurotrophic activity.^79^ A study by a third group indicated gambogic acid increases NGF expression and facilitates bone formation in response to increased osteoblast density induced by mechanical loading in a mouse model.^80^

Gambogic amide is quite cytotoxic (IC_50_ 2.4 μM for HEK293 cells in complete media, not serum-free media as used for cell survival assays); hence, data outlined above were collected for the compound at relatively low doses. It inhibits the proliferation of human umbilical vein endothelial cells (HUVECs; IC_50_ 127 nM) and normal human brain microvascular endothelial cells (NhECs; IC_50_ 174 nM). At 200 nM, it also reduced migration of both HUVECs and NhECs. Gambogic amide inhibits angiogenesis in vitro and in vivo in via a pathway totally independent of TrkA, suggesting off-target effects.^81^ This is important because pharmacological angiogenesis inhibition has led to hypertension, thromboembolism, arrhythmia, heart failure,^82^ hypothyroidism, skin toxicity,^83^ and neurological^84^ issues. Gambogic amide lacks therapeutic efficacy for treatment of traumatic brain injury,^85^ perhaps because this partly depends on angiogenesis.^86^

In general, gambogic *amide* has been studied less than its corresponding *acid*. Gambogic acid is also cytotoxic.^87^ Both the acid and amide were tested in the screen outlined above and demonstrated neurotrophic potential, but only the amide was selected for further studies.^52^

### 2.2 Repurposed Neuroactive Compounds

Compounds in the high-throughput screens described above included the clinically approved antidepressant amitriptyline and deoxygedunin, which has been long appreciated in traditional “folk” medicine. Compounds in this section were identified as potential TrkB agonists and modulators in more focused neuroactive compound repurposing efforts.
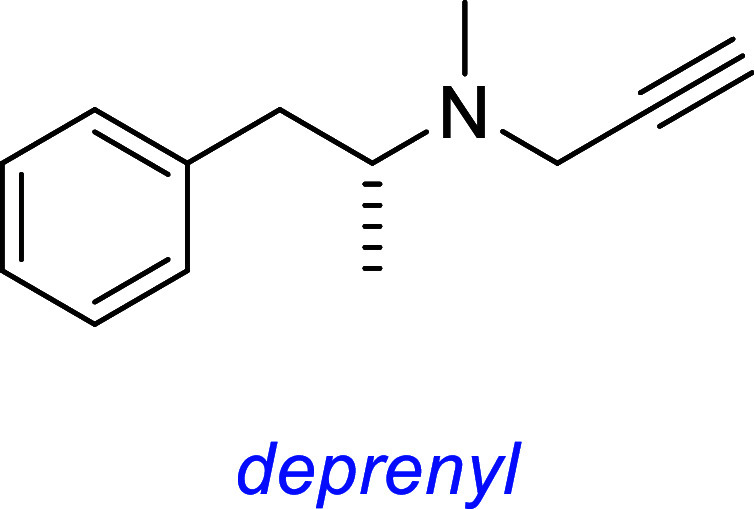


*Deprenyl (Selegiline)* is a selective monoamine oxidase-B (MAO-B) inhibitor approved for treatment of Parkinson’s disease and major depressive disorder.^88^ It elicits neuroprotective-enhanced expression of oxidative-stress-related proteins via the PI3K-Nrf2 pathway. TrkB is a primary upstream activator of PI3K,^89^ and pTrkB was observed by the IP-blotting experiments. The Trk inhibitor K252a inhibited deprenyl-induced PI3K phosphorylation. K252a and a PI3K inhibitor (LY29004) both inhibited deprenyl-induced Nrf2 nuclear translocation. Deprenyl was cytoprotective in cell survival assays and exhibited antioxidative properties in lipid peroxidation assays. Both effects were reduced with coadministration of K252a and LY29004. However, effectors that typically activated pTrkB (phosphorylated AKT, ERK, p38, or JNK) were *not* observed after immunoprecipitation followed by Western blotting. Observation of pTrk but not phosphorylated Trk effectors seems inconsistent to us.

Deprenyl has several mechanisms of action: (1) reducing oxidative radical production, (2) up-regulating activity of superoxide dismutase and catalase, and (3) suppressing nonenzymatic and iron-catalyzed autoxidation of dopamine.^90^ Selegiline does increase the gene expression of all 3 neurotrophic factors (NGF, BDNF, NT-3) in neural stem cells 24 h treatment,^91^ but it does not target Trk receptors or its ligands based on the current literature.
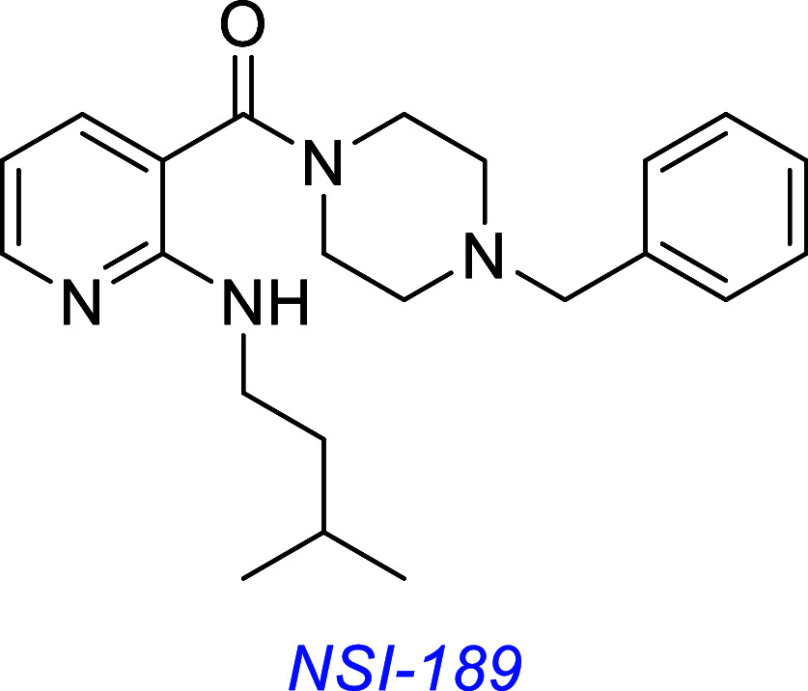


*NSI-189* is a preclinical compound, highlighted in work around 2019. It restores motor and learning function in a mouse model of Angelman’s syndrome (a genetic nervous system disorder). The discoverers reported improvements in synaptic plasticity were mediated by TrkB-AKT pathway activation.^92^ Reported by Alto Neuroscience, Inc. in a press release, NSI-189 is well-tolerated in humans with a favorable safety profile, though it failed to improve symptoms of depressive behavior in phase 2b clinical trials.^93^

### 2.3 Various Leads from Traditional Folk Medicine (Not Necessarily through HTS)

Several traditional medicines have been broadly associated with Trk agonism.
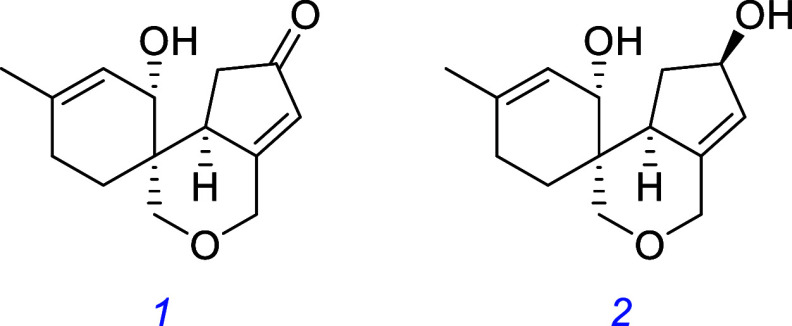


Research on a neurologically active natural product scaffold^94^ led to **1** and **2**, which exhibit neurogenic and neuroprotective effects in ex vivo and in vivo mouse and zebrafish models through a TrkB-dependent mechanism.^95^ 
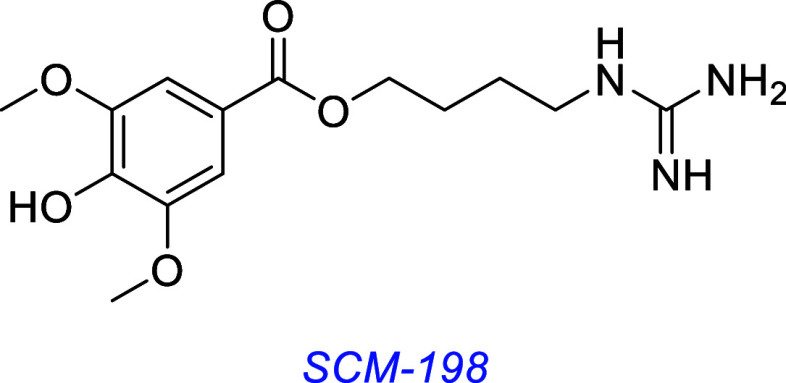


SCM-198, a naturally occurring alkaloid found in Chinese Motherwort, was found to exhibit TrkB-dependent neuroprotective properties in an in vivo mouse model of Alzheimer’s disease.^96^ 
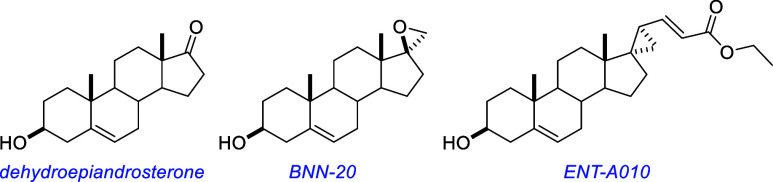


Dehydroepiandrosterone (DHEA) was found to confer neuroprotective effects through interaction with TrkA and p75 in vitro.^97^ The synthetic DHEA derivative BNN-20 ^98^ was found to be neuroprotectant in a mouse model of Parkinson’s disease, inhibiting dopaminergic neuron apoptosis through a TrkB-dependent mechanism.^99^ Another derivative, ENT-A010, protected PC12 cells and DRG neurons from apoptosis in cell survival assays and hippocampal neurons from Αβ-induced apoptosis and ameliorated inflammation in hippocampal microglia in mice.^100^ 
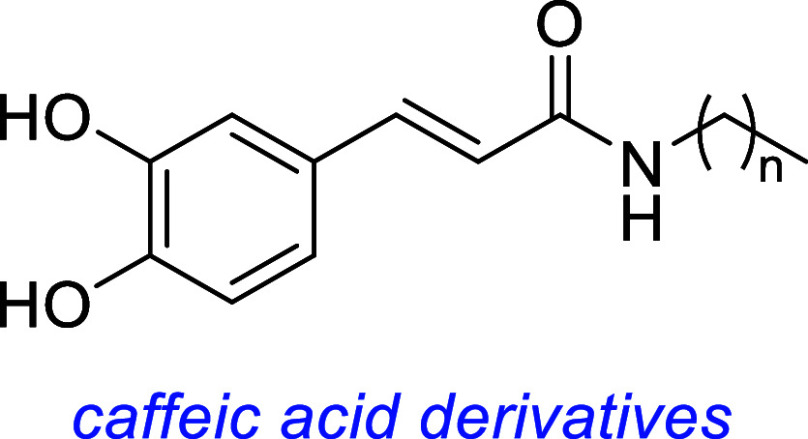


Several caffeic acid derivatives exhibit neurotrophic properties in cell survival experiments conducted in PC12 cells, inducing neurite outgrowth in the presence of NGF, and phosphorylating ERK and AKT, though direct TrkA phosphorylation was undetectable.^101^ 
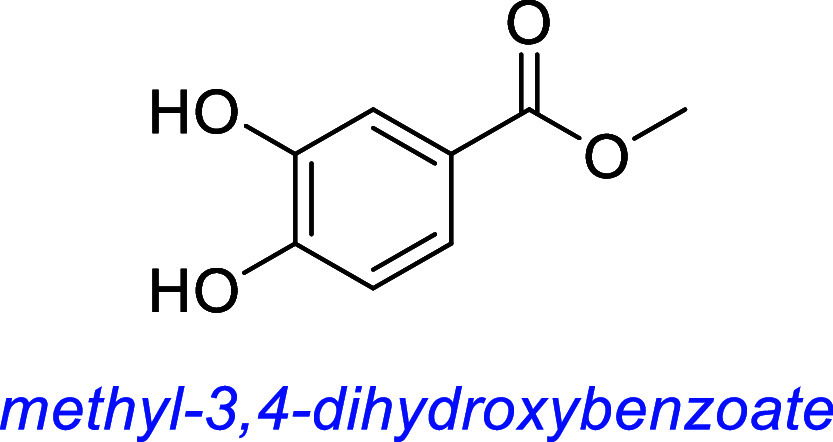


Methyl 3,4 dihydroxybenzoate (MDHB) is patented in China as a potential treatment for neurodegenerative disorders (CN102552233A^102^) and was effective in a mouse model of retinitis pigmentosa, likely through a TrkB-dependent mechanism as it increases the expression levels of BDNF.^103^ 
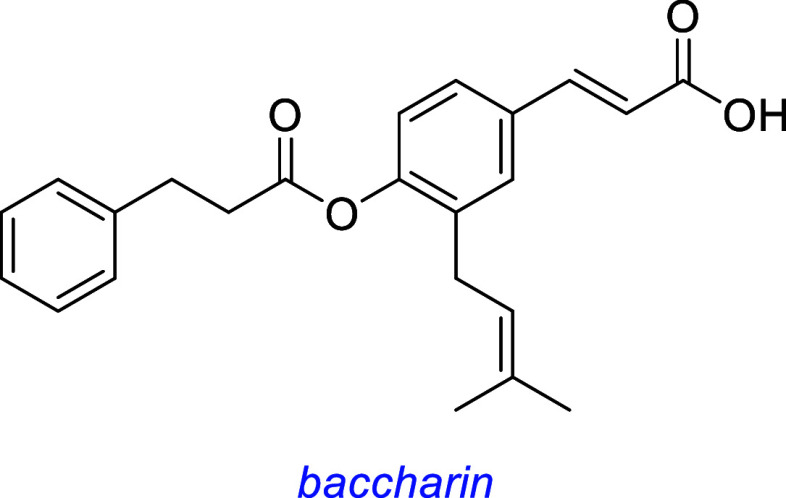


Baccharin, a small molecule isolated from the Brazilian green propolis (a resinous substance produced in the hives of honeybees), exhibits neuroprotective effects in PC12 cell survival experiments, induces neurite outgrowth, and activates TrkA/AKT/MEK1.^104^

## 3 Small Molecule Leads from Computer-Aided Design

Compounds discovered via high-throughput screens are featured in the section above. Here, we distinguish these from other leads originally implicated by structure aided design.

Design of small-molecule neurotrophin mimics can feature computationally based approaches to relate small molecules to the neurotrophin loops and the helix implicated in Trk binding and activation.^105–109^ Compounds from the Longo group, mostly coded “LM···”, are prevalent in this area.
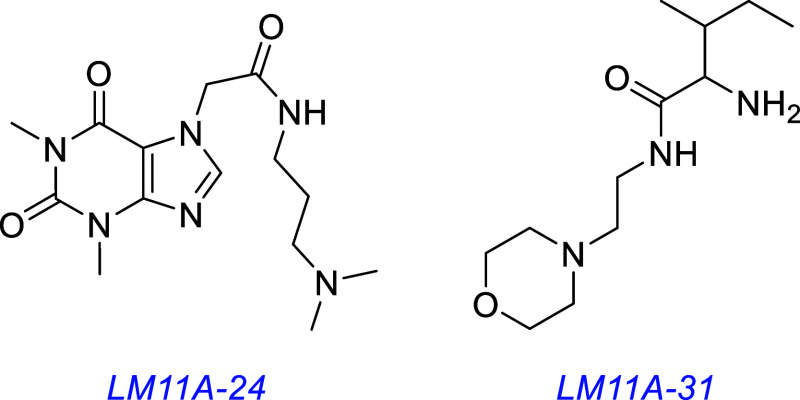


The LM11A series^107,110,111^ were designed in the following way. Simulations on loop (i) of NGF and NT-3 were compared with calculated low energy conformations of small molecules to identify the ones that resemble the peptide loop structures. Pharmacophore features of amino acids in the NT loops (e.g., positive ionizable group, *H*-bond donor, *H*-bond acceptor) were equated to small molecule functionalities with similar characteristics. Over 800,000 virtual compounds were considered, and an average of 35 conformers were generated for each. Hits were filtered using criteria based on visual inspection and accessibility. In an illustrative study,^107^ 23 compounds were obtained and tested, and four were selected (LM11A-7, -24, -28, -31) based on treatment of embryonic mouse hippocampal neurons (partly dependent on supplementation with neurotrophin) and assessment via induced cell survival, immunostaining of downstream phosphorylation, and morphological effects on cells (mostly checking for their continued viability). LM11A-24, -28, and -31 elicited cell survival analogous to that of NGF and BDNF, as evaluated by TUNEL assays to detect DNA fragmentation.

Embryonic mouse hippocampal neurons express TrkB and p75 but not TrkA. Consequently, induced NGF-mediated survival by LM11A-24, -28, and -31 was unexpected because NGF is a prevalent TrkA ligand; the authors suggest p75 binding is involved and tested that hypothesis as described below. In our view, similarities between some of the NT loops are probably less than some of the loop amino acids to small molecule functionality pharmacophore comparisons, so we are not surprised that -24, -28, and -31 activated TrkA, even without p75.

LM11A-24 and -31 were selected for further studies using predictions based on Lipinski oral availability criteria and blood brain barrier (BBB) penetration. Extracellular regions of p75 and TrkA were fused with the Ab Fc region, giving the chimeras p75-Fc and TrkA-Fc. These fusions enabled the adherence to appropriate surfaces bearing anti-Fc Abs. LM11A-24 and -31 inhibited binding of NGF to the p75 chimera in targeted ELISA assays. LM11A-31 was also shown to block the binding of an anti-p75 Ab raised against the neurotrophin binding regions (Ab-9651). This Ab also impaired survival of embryonic mouse hippocampal neurons, implying it blocked a pro-survival function of p75, possibly in a complex with TrkA, further indicating p75-binding.

One of the proteins transmitting intracellular signals from p75 is interleukin 1 receptor-associated kinase (IRAK).^112^ LM11A-24 and -31 induced IRAK association with p75 in PC-12 cells, as monitored via p75 immunoprecipitation, followed by IRAK-targeted Western blotting. These compounds did not induce detectable TrkA or B phosphorylation in hippocampal neuron lysates or in TrkA- or B-expressing NIH3T3 transfectants, as monitored by blotting with Abs raised to detect pY490 or pY515. NFκB AKT activation was also implicated in survival induced by -24 and -31 since this could be nullified by NFκB translocation- and PI3K-inhibitors, whereas an ERK inhibitor did not do this. LM11A-24 and -31 blocked the proNGF-induced cell death of mature oligodendrocytes (measured by TUNEL/MBP staining) and proNGF-p75 association (assayed using the p75·Fc chimera mentioned above).

In subsequent work, LM11A-24 and -31 were shown to prevent p75^NTR^-dependent motor neuron death^113^ and improve in vitro neuron survival in models of Alzheimer’s disease.^114^ LM11A-31 also exhibited effects in in vitro primary neuron models of anticancer drug-induced neurotoxicity^115^ and feline immunodeficiency virus (FIV),^116^ and it prevented neurite degeneration and cognitive deficits in a mouse model of Alzheimer’s disease.^117^ Another group found LM11A-24 protects retinal ganglion cells in mouse models of retinal injury via inhibition of the NGF·p75^NTR^ interaction.^118^ A second group using ^11^C labeled -24 found it influenced firing in neurons expressing p75 ex vivo, but it performed poorly as a PET radiotracer to map p75 expression in vivo.^119^

A 10 day European Union safety and tolerability clinical trial was conducted on LM11A-31-BHS, and it raised concerns. “31” increased γ-glutamyl transferase (an early predictive marker for cardiovascular and renal diseases^120^) in 1 out of 8 participants but none in the placebo group. The majority (5 out of 8) of participants suffered headaches with LM11A-31-BHS, none in the placebo group.^121^ It is difficult to determine causal relationships using such a small sample size, but LM11A-31 also entered a larger clinical trial. This resulted in 16 of the 242 participants discontinuing treatment due to adverse events such as a dose-dependent increase in gastrointestinal issues, transient asymptomatic eosinophilia, and nasopharyngitis. The report states adverse events were more frequent in groups receiving higher doses.^122^ This phase II clinical trial ended in 2020,^123^ and no efficacy or safety data have been published yet; we do not know if clinical development of LM11A-31-BHS will go further.
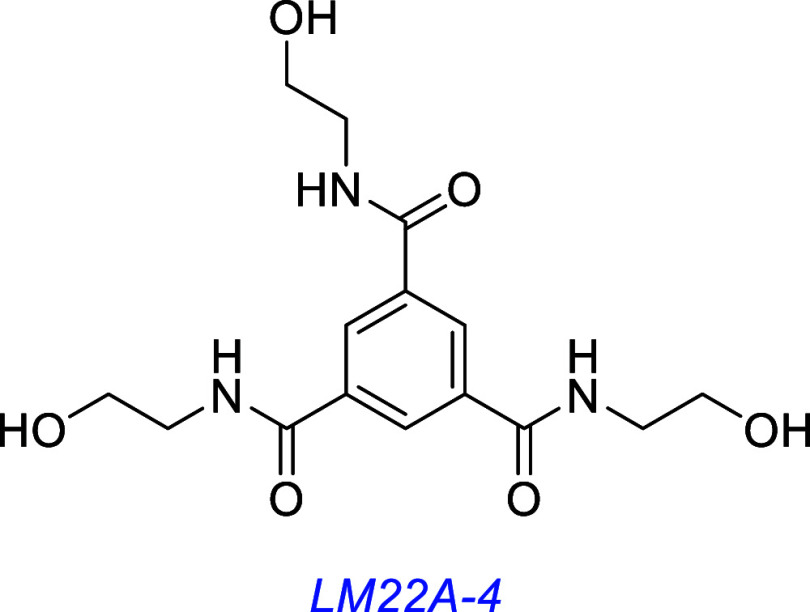


Another series, LM22A compounds, were designed to mimic loop (ii) of BDNF.^108^ Five hits promoted E16 mouse hippocampal neuron survival, and of these, LM22A-1 and -4 were evaluated further. They induced survival of the hippocampal neurons at 80–90% the activity of BDNF with EC_50_s between 200 and 500 pM.

However, other experiments indicated that BDNF was *not* associated with LM22A compounds. Thus, their survival-inducing activity was not affected by coincubation with an anti-BDNF Ab, indicating BDNF was not required and BDNF upregulation was not induced. On the other hand, LM22A-1, -2, -3, and -4 were inferred to act through TrkB since coincubation with the pan Trk kinase inhibitor K252a (200 nM) diminished induced cell survival. Similarly, coincubation with an anti-TrkB-extracellular domain antibody decreased survival mediated by the sample or by BDNF. All four compounds were evaluated by Western blotting using an antipan-phospho-Trk pY490 antibody using NIH3T3 transfectants; phosphorylation of TrkB, but not of A or C, was observed.

LM22A-4 was selected for further study because of its structural simplicity and amenability to chemical modification. It was active in cell survival of NIH-3T3-TrkB transfectants but not -TrkA, -C, or -p75 ones. Binding to the TrkB extracellular domain was implicated in affinity assays featuring the TrkB-ECD·Fc chimers on beads (LCMS/MS quantification of bound LM22A-4, blocked by BDNF binding). Competition between LM22A-4 and TrkB-ECD·Fc-Cy3 was measured via fluorescence polarization, and it proved to be a competitive inhibitor (IC_50_ 47 nM). Specificity was also investigated for Trk- or p75-expressing transfectants via fixed-cell immunoassay targeting assays; LM22A-4 inhibited BDNF·TrkB interactions but not NGF·TrkA, NT-3·TrkC, or BDNF·p75.

LM22A-4 induced pAKT and pERK much more robustly than pTrkB with kinetics like BDNF in hippocampal neurons and in NIH3T3-TrkB cells (but not in the TrkA- and TrkC-expressing transfectants). This pERK was inhibited by coincubation with anti-TrkB antibodies or K252a. Co-incubation of LM22A-4 or BDNF with MAPK and PI3K inhibitors reduced neurotrophic activities in hippocampal neurons, providing further evidence for signaling through TrkB. TrkB, AKT, and ERK were robustly activated by LM22A-4 in vivo in mouse hippocampal and striatal tissue following daily intranasal administration over 7 days. LM22A-4 prevented neuronal cell death with similar efficacy to BDNF in several in vitro models of neurodegenerative diseases, including Αβ-induced cell death in hippocampal neurons, MDD^+^-induced cell death in SH-SY5Y cells (a validated Parkinson’s model), and quinolinic acid-induced cell death in striatal neurons (a model of Huntington’s disease). In each of these experiments, LM22A-4 and BDNF efficacies were inhibited by coadministration of K252a, indicative of a Trk dependence. A test panel of 57 receptors (Cerep Inc.) were used to screen LM22A-4 selectivity; no significant binding to any was detected.

Whatever its mechanism, LM22A-4 beneficially affects neuronal function in vivo. It promoted recovery of motor learning function in a rat model of TBI (though total lesion volume was unaffected). It facilitated recovery after a hypoxic–ischemic stroke in mice^124^ and in mouse models of Rett syndrome,^125–127^ Huntington’s disease,^128^ optic neuropathy,^129^ and myelin sheathe repair.^130,131^ It has also been found to regulate differentiation of calcium secreting cells (cementoblasts) through a TrkB-AKT-ERK-dependent mechanism.^132^

In summary, a large body of evidence, mainly, but not exclusively, from Longo’s lab, indicates LM22A-4 modulates TrkB activity. However, despite these widespread reports, some groups have questioned if LM22A-4 activates TrkB. These contrarian reports are discussed below.^69,70^ 
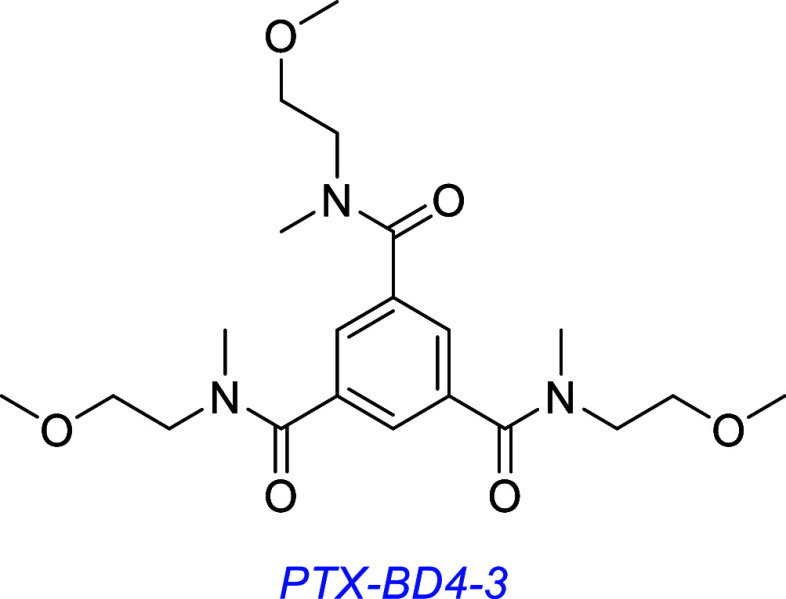


A derivative of LM22A-4, PTX-BD4-3, reduces epileptogenic activity in a model of post-traumatic epileptogenesis in mice,^133^ induces hippocampal neuron survival in vitro, and improves motor learning deficits in an in vivo mouse model of Rett Syndrome.^134^ 
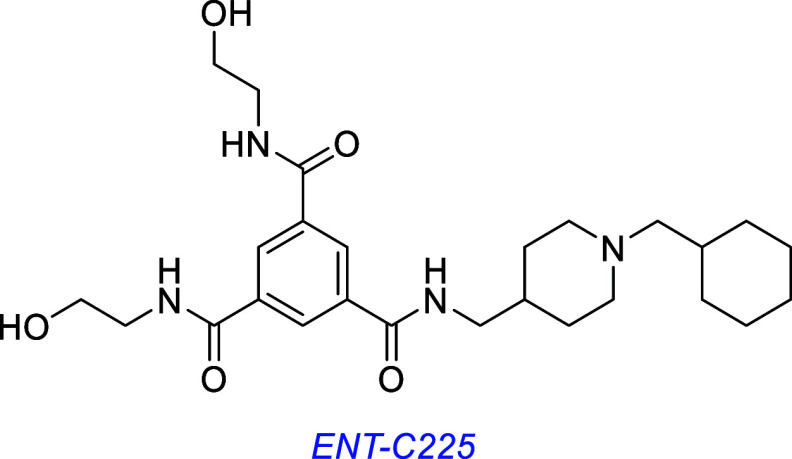


Later, several LM22A-4 derivatives were found to induce pTrkB and pAKT and induce neurite outgrowth in SH-SY5Y cells; ENT-C225 was found to be most active.^135^ 
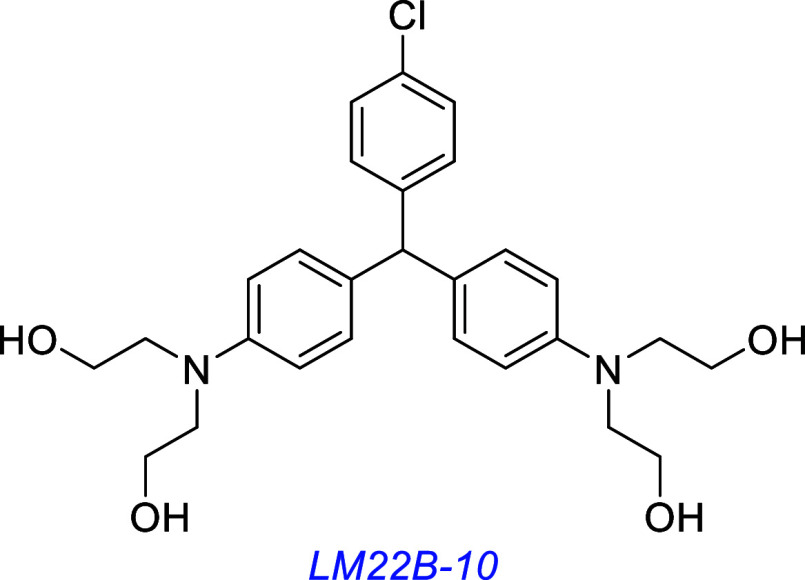


LM22B-10 ^109^ was discovered by expanding the virtual library size to over 5 million compounds. Four out of 18 compounds tested in a primary hippocampal neuron survival screen were active, and -10 was the one selected for further study. Considerable evidence indicates LM22B-10 activates TrkB and C. Thus, it induced the same level of neurite outgrowth as BDNF or NT-3 and competed with them for binding the TrkB and C extracellular domains (LCMS/MS analyses and fixed-cell binding assays). LM22B-10 induced the survival of NIH3T3-TrkB and TrkC transfectants regardless of p75 expression, whereas activities of BDNF or NT-3 in the same assay were decreased by p75 expression. It caused phosphorylation of TrkB, AKT, ERK, and PKC but to a much lower degree than BDNF in TrkB-expressing cells; however, its kinase activity observed via TrkB immunoprecipitates was significant. Effects of -10 on the TrkC receptor activation pattern and downstream signaling were like NT-3 in TrkC transfectants. It showed minimal off-target receptor activation in the Cerep Inc. panel test.^109^

LM22B-10 is active in vivo in models of neuronal diseases. It activates TrkB, TrkC, AKT, and ERK in vivo and increases levels of proteins typically upregulated in TrkB or C activation (e.g., synaptophysin and PSD95) in brains of aged (15–17-months) male mice and dendritic spine density in their hippocampal neurons. A different group found LM22B-10 effective in restoring pERK levels and synaptic plasticity in vivo and behavioral normalcy to a rat model of Cacna1c gene hemizygosity (a gene in which variation is strongly linked to psychiatric disorders like schizophrenia and BPD).^136^ A third group found it activated ERK and CREB in primary mouse DRG-CSC neuron coculture and was effective in nerve regeneration and wound healing in an in vivo model of corneal injury.^137^ LM22B-10 was found to alleviate striatal and motor function deficits in a mouse model of Huntington’s disease.^138^ 
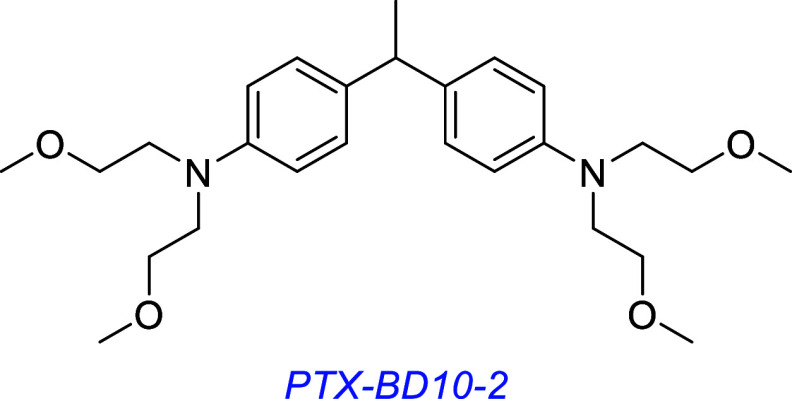


A LM22B-10 derivative, PTX-BD10-2, prevented cholinergic neuron atrophy in an in vivo model of advanced Alzheimer’s disease.^139^

PTX-BD10-2 is also in preclinical development for Alzheimer’s disease. It has demonstrated strong neuroprotective effects, suggesting a therapeutic potential for neurodegenerative disease. However, PTX-BD10-2 does have safety concerns because it inhibits four hepatic cytochrome enzymes, including the inhibition of CYP2C9 by 72%.^139^ CYP2C9 is required for metabolism of endogenous molecules and drugs, and inhibition of this induces pathogenesis in patients.^140,141^ Otherwise, there are promising preclinical data for the use of PTX-BD10-2 for Alzheimer’s disease.
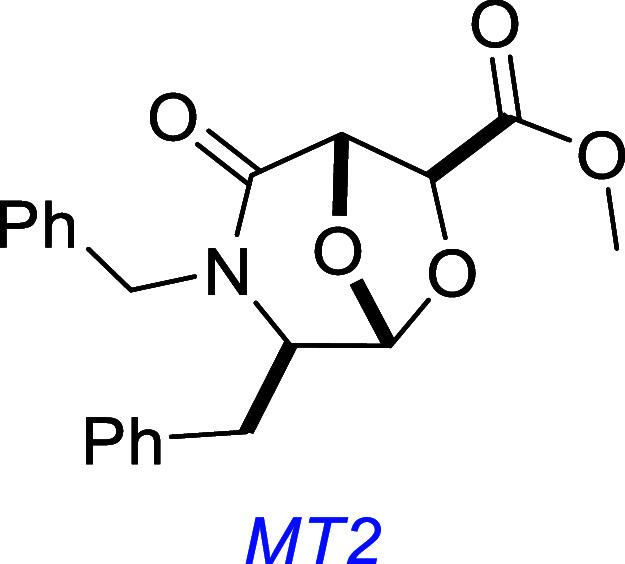


Independent of small molecules discovered by Longo, MT2, a bicyclic scaffold presenting two amino acid side chains, was computationally designed to fit the TrkA D5-binding pocket and was shown to activate TrkA by in vitro blotting and cell survival experiments.^142^ 
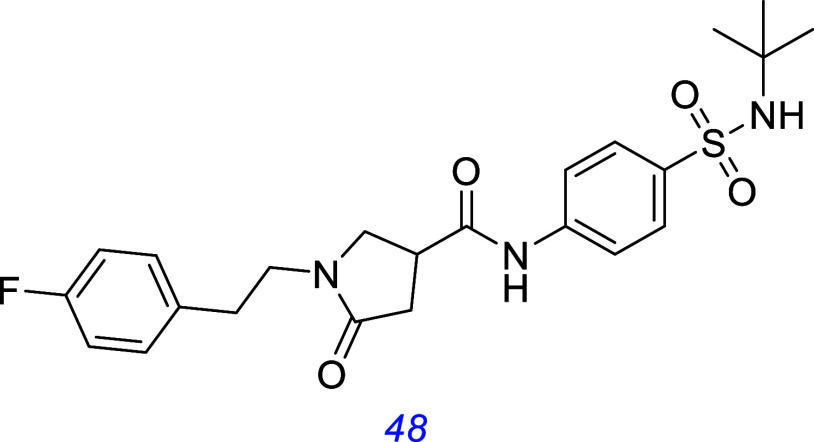


To identify potential TrkB agonists, another group identified **48** using an in silico design strategy to target the BDNF binding interface of TrkB. It was found to have TrkB activity in cell proliferation assays and blotting and performed well in a forced swim test in mice designed to observe potential antidepressant effects.^143^

## 4 Contradictory Reports: Do Trk Agonists Work As Reported?

Several groups published data suggesting small-molecule Trk modulators described above do *not* function primarily through Trks or their mechanisms of action are more complex than they appear. Based on relatively numerous reports described above, there can be little doubt many of the putative Trk agonists are active in vivo; the question is how. Unlike most of the other sections in this review, this one discusses many small molecules simultaneously, so Figure 3 summarizes some interesting small molecules from the discussions up to this point, as s reminder of their structures; structural features become increasingly important for the rest of this review.

**Figure 3:**
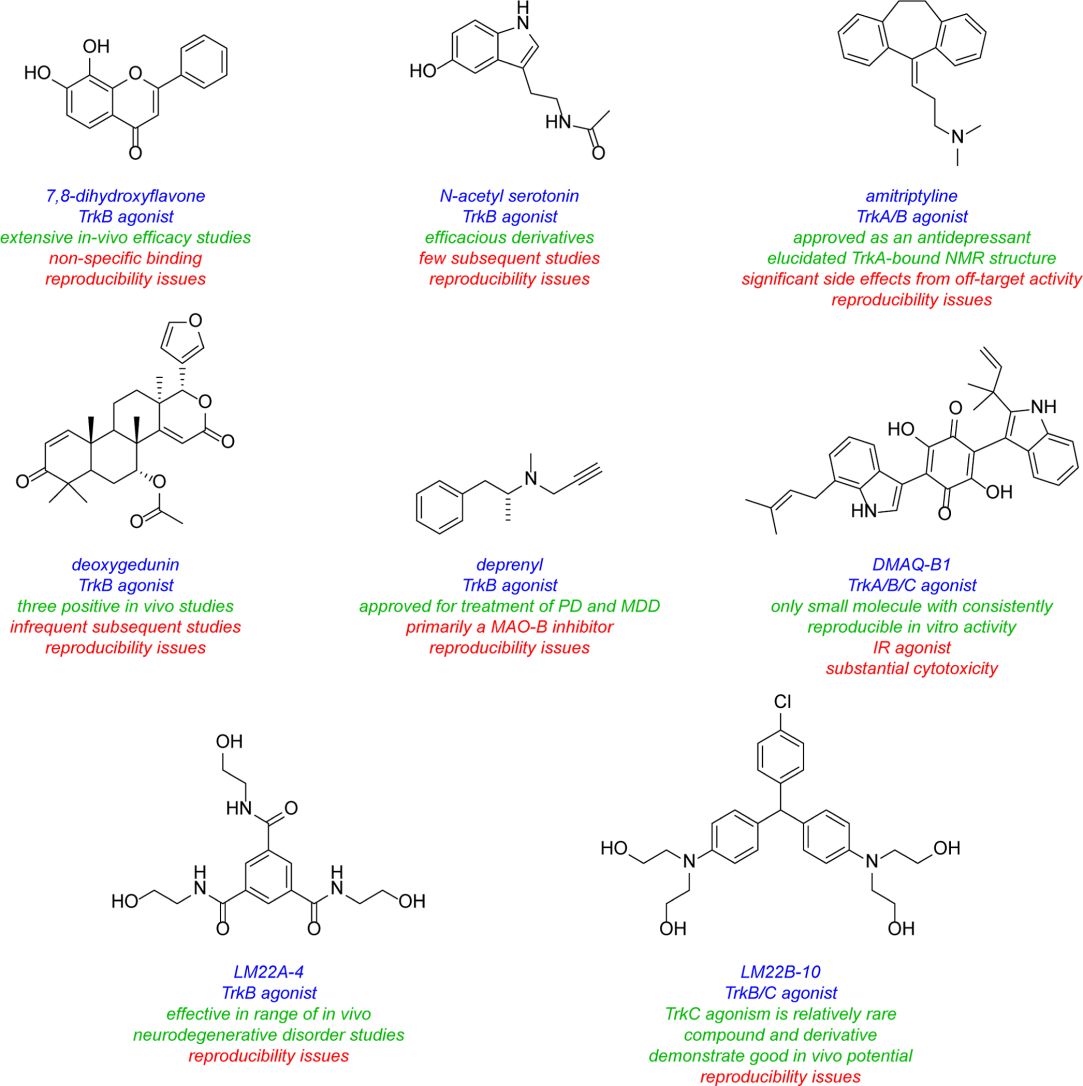
Most widely studied small molecule leads for Trk agonism. Reported Trk selectivities in blue, outline of observations in green, and potential issues in red.

Groups disputing some putative Trk agonists have independently tested their potential to signal through the Trk receptor. First, Bard and co-workers in 2014 compared two TrkB Abs, with five putative TrkB agonists reported by others: 7,8-DHF, amitriptyline, and LM22A-4 (these three overlap with Sames and others below) and NAS. In 2017, Sames’ group^70^ investigated Trk activation by six putative TrkB agonists based on biochemical studies: 7,8-DHF, amitriptyline, LM22A-4 (these like Bard), deprenyl, DMAQ-B1, and deoxygedunin, and then applied the validated assays to screen a diversity-oriented synthesis (DOS) library of 40,000 compounds with ultimately few hits. In 2021, Matłoka’s group reported high-throughput screens on about 1000 compounds including several reported as TrkB agonists.^144^

### 4.1 Assays to Detect Phosphorylation in Cells

Sames’ group attempted direct blotting assays based on anti-pTrk antibodies using HEK293 TrkB transfectants and primary cortical neurons. They found these blotting assays to be unreliable and suggested several reasons. First, they depend on the Ab quality. Suboptimal selectivities for many commercial anti-pTrk Abs can result in multiple bands, effectively giving high noise. pTrk Abs are not used as widely as ones for more common targets and hence are not subjected to as extensive quality control by the customer base. None of the series, 7,8-DHF, deprenyl, amitriptyline, LM22A-4, DMAQ-B1, or deoxygedunin, showed TrkB agonistic activity.^70^

Bard and co-workers also Western blotted cell lysates of treated cells for pTrkB (using anti-pY515 Ab), pAKT, or pERK; the cells used were as in the first assay described below (“TrkB-CHO CellSensor”). No phosphorylation was observed for 7,8-DHF, amitriptyline, NAS, and LM22A-4.^69^ On the contrary, NAS and LM22A-4 suppressed baseline levels of pERK relative to the DMSO control; the authors suggested this may be due to modulation of other proteins and/or cytotoxicity.

Matłoka reported 7,8-DHF, 7,8-DHF, LM22B-10, 7,8,3′-THF, 4′-DMA-7, 8-DHF, GSB-106 (a peptide, see below), LM22A-4, HIOC, NAS, and NSI-189 were unable to induce phosphorylation of TrkB, Akt, and Erk1/2 in conventional blotting assays.^144^ However, DMAQ-B1 was observed to induce statistically significant pTrkB, pPLCγ, pAKT, and pERK1/2, consistent with earlier reports.^75^

Enzyme linked fixed cell immunoassays (ELFI, Figure 4) have several advantages relative to pTrk blotting.^70^ First, higher throughput is possible than in conventional blotting because only fixed cells are used; this is important because many experiments are required to obtain satisfactory statistical significance for measuring weak activation. Second, ELFI detects activation indicators downstream of Trk’s. Downstream phosphorylated kinases for TrkB include pAKT and pMAPK, and Abs for these targets are used extensively and hence solidly validated. Sames’ used two positive neurotrophin controls (BDNF and NT-4), zinc pyrithione (a compound that results in activation of the kinase domain of TrkB intracellularly via SRC) and the same cell lines used in his blotting assays: HEK293 TrkB transfectants and primary cortical neurons. 7,8-DHF, deprenyl, amitriptyline, LM22A-4, and deoxygedunin showed no pAKT and pMAPK activity in either cell line. Only DMAQ-B1 induced pAKT and pMAPK, and this was active for both cell lines.

**Figure 4:**
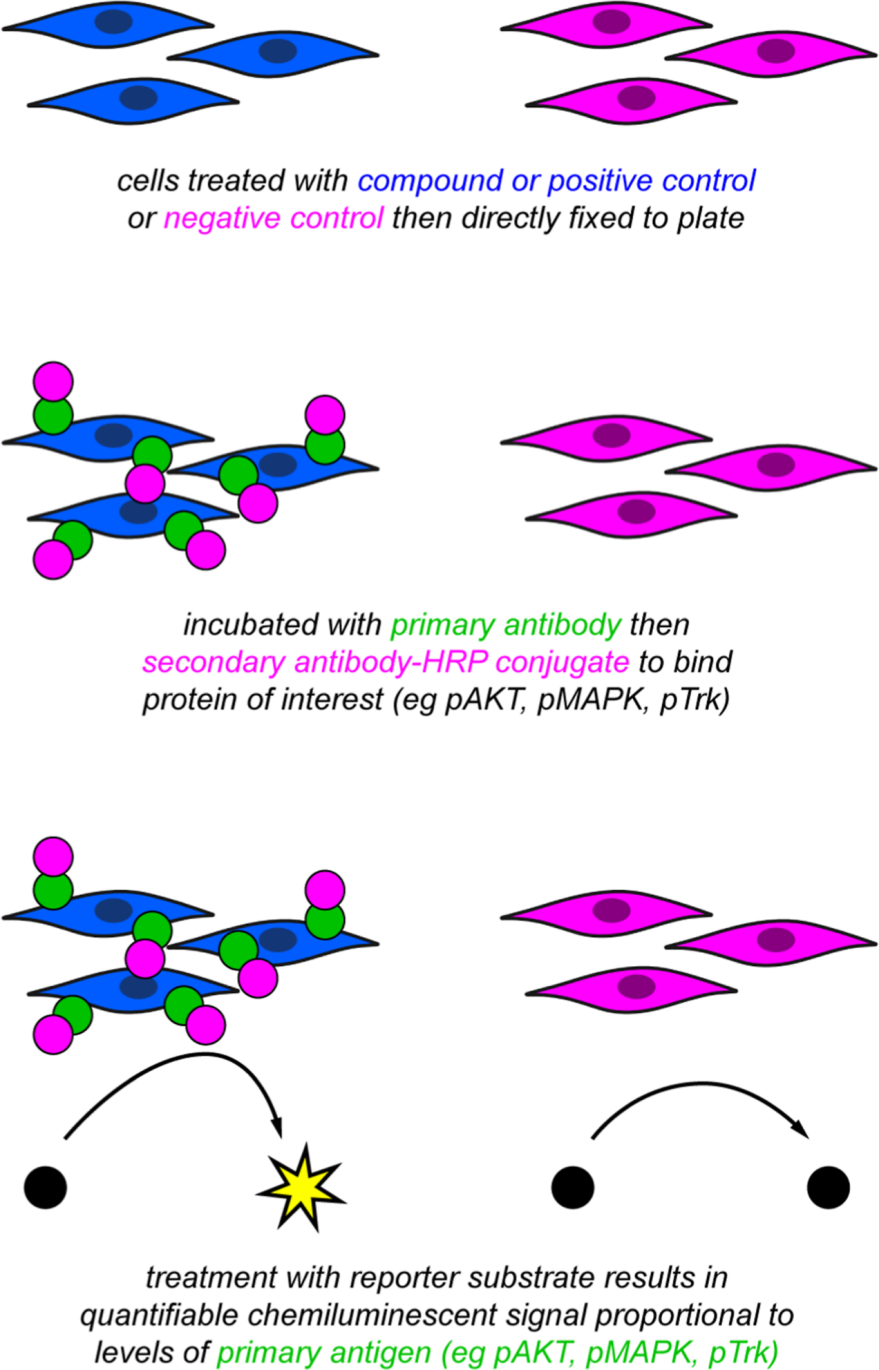
Workflow of the enzyme-linked fixed-cell immunoassay.

Sames also used ELISA featuring supported anti-Trk Abs targeting an unspecified extracellular epitope in the same cell lines.^70^ Anti-TrkB Abs tend to be more reliable than the ones detecting phosphorylated forms, anti-pTrkB, used in the blotting assays described above. Lysates from sample-treated cells may contain pTrkB, which is captured on a plate via anti-TrkB binding the extracellular domain, and then analyzed with a second Ab-HRP that broadly detects pTyr. DMAQ-B1 induced weak but detectable signals for pTrkB, but no response was observed for primary cortical neurons. 7,8-DHF, deprenyl, amitriptyline, LM22A-4, and deoxygedunin did not give detectable responses in this assay.

### 4.2 Other Cellular Assays

7,8-DHF is the most widely studied of the putative TrkB agonists; Matłoka and co-workers selected this for their cell survival studies.^144^ DMAQ-B1 and 7,8-DHF were not able to induce cell survival of differentiated TrkB expressing SH-SY5Y cells in their experiments.

Bard tested 7,8-DHF and LM22A-4 in a primary rat cortical and striatal neuron coculture system, wherein mutant huntingtin was transfected to induce an effect resembling Huntington’s disease. This is a specialized cell survival assay. BDNF, the positive control, rescued from cell death and generated pTyr. 7,8-DHF and LM22A-4 did not induce these outcomes, and 7,8-DHF was cytotoxic at concentrations above 20 μM in this system.

In 2011, Chen et al. reported 7,8-DHF protected against glutamate-induced toxicity in mouse hippocampal-derived HT-22 cells. These do *not* express TrkB, and it was hypothesized that the cytoprotective effects observed came from the flavonoid’s antioxidative properties.^145^ Consistent with this, they showed that 7,8-DHF stimulates increased production of glutathione.

### 4.3 Binding Assays

Matłoka’s group used microscale thermophoresis to screen binding to the isolated, solubilized, extracellular domain of TrkB.^144^ Nearly 1000 compounds were tested, including many of the Trk agonists reported here. Binding was detected for 7,8-DHF, DMAQ-B1, and LM22B-10 (*K*_d_ values of 1.3, 5.6, and 83 μM). Other putative TrkB agonists did not give detectable *K*_d_ values, including 7,8,3′-THF, 4′-DMA-7, 8-DHF, GSB-106 (a peptide, see below), LM22A-4, HIOC, NAS, and NSI-189.

Matłoka’s *K*_d_ for 7,8-DHF·TrkB (*K*_d_ of 1.3 μM) contrasts to the *K*_d_ of 320 nM for the isolated purified extracellular TrkB domain reported by Ye using radiolabeled 7,8-DHF.^56^ In our view, this is unsurprising because such assays are highly dependent on the preparation of the receptor fragment. However, this group also found 7,8-DHF bound additional 133 different intra- and extracellular protein targets, highlighting the lack of selectivity and potential for a wide range of Trk-independent effects.

### 4.4 Assays Featuring Cells Engineered to Couple Trk Activation with Detectable Signals

Sames and Bard both used the DiscoverX PathHunter Assay and TrkB-CHO CellSensor assays to screen putative Trk modulators.^69,70^ One of these assays featured the Invitrogen CHO CellSensor TrkB-NFAT-β-lactamase encoding gene, involving the separation of a FRET (Forster resonance energy transfer) pair upon β-lactamase expression. The other involves complementary fragments of β-galactosidase linked to the Trk intracellular domain and the SHC1 adaptor (DiscoveRx PathHunter); binding of the modified SHC1 adaptor protein to the modified Trk intracellular domain reconstitutes β-galactosidase. This enzyme processes a proprietary substrate added to the assay, eventually leading to chemiluminescence. Both these were validated for BDNF as a positive control, but none of the *featured* small molecules screened by Sames^70^ (7,8-DHF, deprenyl, amitriptyline, LM22A-4, and DMAQ-B1, and deoxygedunin) and Bard^69^ (7,8-DHF, amitriptyline, NAS, and LM22A-4) showed TrkB agonism. The screening of the DOS library with the DiscoveRx PathHunter assay resulted in 83 hits, of which three were active in the CellSensor assay, though none were active in the ELFI or ELISA.^70^

### 4.5 Negative Findings in Advanced In Vivo Assays

Negative in vivo studies do not prove anything definitively, but the following report is interesting insofar as the authors rationalize why a putative TrkB agonist did not work. Zaitoun et al. used 7,8-DHF in a mouse model of oxygen-induced ischemic retinopathy (OIP) and found it did not prevent retinal vessel obliteration or retinal neovascularization any better than a negative control.^146^ These processes would be expected to be perturbed by the TrkB agonism. They hypothesized that this could be due to downregulation of hypoxia-induced transcription factors induced by high oxygen levels, resulting in lower expression of important cytokines such as VEGF, which can result in inhibition of TrkB phosphorylation. Further, the TrkB.T1 isoform, lacking the catalytic kinase domain (though full-length TrkB was also expressed), was shown to be the predominant variant of TrkB expressed in the retinas of the OIR model mice, which could explain the lack of apparent TrkB signaling.

## 5 TrkB Positive Allosteric Modulators (PAMs)

Allosteric modulation features depression or enhancement of receptor activity as a result of ligand binding to sites not overlapping with that of the endogenous ligand. Positive Allosteric Modulators (PAMs) binding in this way leads to enhanced activities.

PAMs of the Trk receptors are not true agonists, but recent developments in this area are relevant to the material presented above and influence how it should be interpreted. Many of the key findings on TrkB PAMs come from two pivotal papers^147,148^ from a team featuring Castrén and several other senior investigators.

In *Cell*,^147^ they report the TrkB transmembrane region has a cholesterol recognition sequence,^149^ which they may have been the first to recognize in Trk receptors. This is characterized by the consensus (CRAC) domain, or its inverted version CARC, which aligns parallel to cholesterol in the membrane. BDNF-induced neuroplasticity was known to be cholesterol-dependent, but reasons for this were not known up until this point. They also knew that regions of the synapse near the cleft tend to have higher cholesterol concentration, and the significance of that also becomes clear. In their work, the team proved that BDNF activity was increased with cholesterol up until about 20 μM but was suppressed at higher concentrations. This is significant because the affinity of cholesterol for the TrkB cholesterol recognition sequence was also around 20 μM.

Their investigations were greatly facilitated by a negative control cell line bearing a TrkB.Y433F mutant. This mutant features substitution of a tyrosine known to be important for CARC·cholesterol interactions in general, with phenylalanine, which negates binding. BDNF binds to the mutant extracellular region, but the level of phosphorylation of TrkB.Y433F in response to treatment with BDNF was diminished. Further, the mutation suppressed migration of the TrkB.Y433F complex with BDNF to lipid-raft regions, where the native complex signals most efficiently. Molecular dynamics simulations indicated TrkB adopts an “open-crossed conformation” in the absence of cholesterol, where the CARC domain and Y433 are exposed, but a “closed-cross” (our terms) in the presence of optimal cholesterol, which they attribute to the active conformation (Figure 5a). Subsequently, they added to their interpretations in a review.^148^ They suggest the TrkB at the synaptic cleft exists as active crossed dimers, in equilibrium with parallel receptor conformations, which are readily absorbed into intracellular vesicles and hence shielded from BDNF. Cholesterol stabilizes the active crossed conformation in lipid rafts and increases TrkB effective concentrations and residence times of functional TrkB at synaptic cleft surfaces.

**Figure 5:**
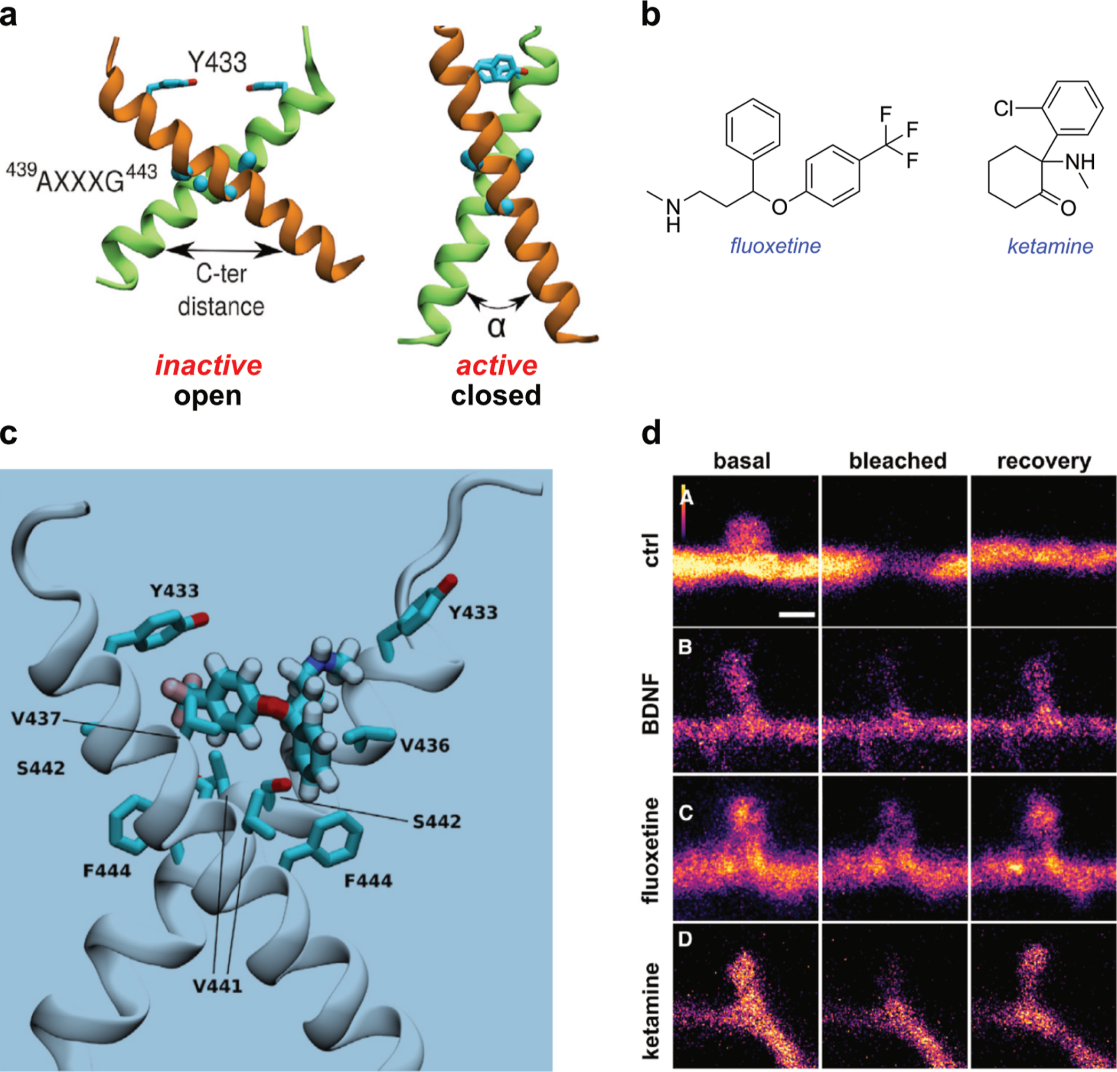
(a) Molecular modeling of the inactive open conformation, and the active closed one. AXXXG is the region where the two helices contact each other. (b) Structures of the featured antidepressants. (c) Fluoxetine modeled in the crevice between the TrkB transmembrane helices. (d) Rat hippocampal neurons transfected with TrkB.GFP immediately after photobleaching (center) and 2 min later (right): A untreated, B BDNF-treated, C fluoxetine-treated, or D ketamine-treated. Created by modifying open source graphics in the featured *Cell* paper, https://doi.org/10.1016/j.cell.2021.01.034.^147^

Binding of various antidepressants, notably fluoxetine and ketamine (Figure 5b), to TrkB were explored. Both were shown to be low micromolar binders of TrkB (∼1–3 μM). Neither competed with BDNF, as expected for compounds that bind in the transmembrane, and not the extracellular, domain. Cholesterol *enhanced* TrkB interactions with fluoxetine and ketamine, indicating separate but synergistic binding sites. Modeling indicated that fluoxetine binds in a cleft between the two TrkB dimers, stabilizing them in the putative active crossed conformation (Figure 5c).

Photobleaching of GFP·TrkB in primary hippocampal neurons, followed by observation of recovery (via fluorescence recovery after photobleaching, FRAP), showed fluoxetine and ketamine promoted trafficking of the receptor into dendritic spines (5 days), indicative of neuroplasticity. GFP·TrkB.Y433F-transfected cells did not show this effect.

This group also performed a series of in vivo experiments to probe BDNF-induced plasticity in wild type mice (hence confirmed what had been observed previously) and compared with parallel studies on mice engineered to express TrkB.Y433F. Only the wild type mice showed evidence of enhanced neuroplasticity, indicating a functional cholesterol binding site is essential. They also confirmed the antidepressants increase proliferation of newly born dendrite neurons, as had been reported, but not in heterozygous mice bearing the TrkB.Y433F mutation.

In a water maze test, fluoxetine and ketamine promoted long-term potentiation (LTP) in wild type mice and in ones without the appropriate serotonin receptor for this drug (5HTT.ko). Enhanced LTP was not observed, however, for mice with the TrkB.Y433F mutant.

Overall, their data implicate low micromolar binding of the featured antidepressants near, but not overlapping with, the cholesterol binding domain of the TrkB transmembrane region. This binding promotes neuroplasticity, which would be desirable in patients needing to form alternative neural networks after an event or an environment causing depression or trauma. Consistent with this, regular dosing of antidepressants for weeks tends to be necessary for the observation of clinically beneficial effects. This observation is interpreted as follows: time and serial dosing are required for cumulative drug accumulation in the brain to reach concentrations required for significant binding to the TrkB receptor.

This team notes Trks A and C do not have transmembrane cholesterol binding domains. Nevertheless, some experiments were performed using TrkB·TrkA.TMD chimeras, and indeed, similar effects were *not* observed. Most of the compounds discussed in this review are putative TrkB agonists, not A or C, and how these observations may be related is discussed in the Conclusions section.

Evidence has been emerging for decades indicating psychedelics, including LSD (lysergic acid), may have beneficial effects for treatment of depression and trauma, even in extreme cases like posttraumatic stress disorder.^150^ For instance, clinical trials featuring LSD were reviewed in 2020, and it is widely accepted that low doses of LSD and other substances in this class have antidepressant effects.^150^ Further, TrkB and BDNF were known to be necessary for psychedelic-induced neuroplasticity, though the mechanism was not established as of 2022.^151^

Castrén’s team’s work in *Cell* raised the possibility TrkB, rather than serotonin receptors, is associated with the antidepressant activity of some psychedelics, especially when supported by therapy, environment change, or mindfulness techniques to support neural reprogramming.^152,153^ Thus, the team investigated effects of psychedelics on TrkB in the second pivotal paper, which was published in *Nature Neuroscience.* ^148^ They collected abundant evidence LSD and some other psychedelics are TrkB PAMs which bind near the transmembrane cholesterol binding site. An outline of their studies follows.

They found tritium-labeled LSD binds the TrkB transmembrane domain with *K*_d_s < 1 nM (human, rat, and mouse), i.e., 1000× more strongly than fluoxetine and ketamine (see above). This binding is impaired by TrkB.Y433F cells, the same mutation that negated binding of the antidepressants. Similar binding was *not* observed in the TrkB·TrkA.TMD chimera. Long residence times were noted for wild-type (wt) TrkB·LSD. No other known targets were found for LSD binding in proteomic mass spectrometry, suggesting that TrkB is its primary target.

Another psychedelic, psilocin (PSI), also binds the same site in the TrkB intracellular domain with low nanomolar affinities (via competition experiments to determine *K*_i_s). Other competition and mutation experiments showed the LSD/PSI binding site is distinct from the one that binds fluoxetine and ketamine.

The following experiments were performed using LSD and PSI, “the psychedelics” unless otherwise stated. They increase TrkB neuronal surface retention and promote interaction with a raft-restricted kinase (Fyn), indicating localization on raft-like membrane structures at the synapse.

The psychedelics *increase* pTrkB induced by BDNF, and signaling is downstream of that receptor. This is also true in cells that do not express the 5-HT_2A_ receptor, suggesting a mechanism of action independent of the serotonin receptors. LSD also potentiated very low concentrations of BDNF with respect to induced pTrkB. All these observations are consistent with stabilization of the TrkB dimerization by the small molecules. LSD induced selective phosphorylation of the TrkB receptor at Y816 in hippocampal and cortical cultures after a single administration to live mice. That is interesting because pY816 interacts with phospholipase C gamma 1 (PLCγ1), and this kinase is known to regulate intracellular calcium signaling and antidepressant effects. They showed TrkB·PLCγ1 in the prefrontal cortex and hippocampus of wild type mice was increased by LSD in wt, but not Y433F, mice, though total TrkB expression was unchanged. Microscopy experiments confirmed the psychedelics increased TrkB·PLCγ1 in dendritic spines. FRAP experiments similar to those in Figure 5d featuring GFP-tagged TrkB in neuronal cultures showed rapid diffusion of the receptor into dendritic spines of wt but not Y433F cultures. Similarly, neurite outgrowth and spine formation were only observed in the wt cultures treated with the psychedelics.

Extensive studies of behavior and neuroplasticity in mice were performed. Throughout, all indications were that the Y433F mutation in heterozygous mice did not interfere with BDNF function but negated the effects of the psychedelics. This is consistent with normal function of BDNF, *enhanced* in the presence of the psychedelics, but only if there is a suitable transmembrane binding site. LSD was found to *double* the number of surviving dentate granule cells (DGCs) in the hippocampus 4 weeks after one administration. Another test for neuroplasticity is to measure the shift in ocular dominance toward the open eye in mice deprived of vision of the other eye; LSD promoted this. LSD also promoted antidepressant effects in wt mice (only) as measured by placing them under chronic stress by repeated force swimming sessions. Similarly, 3 days after a conditioned fear response, a single dose of LSD elicited a decrease in behavioral freezing, but only after extinction training. This is consistent with observations in humans that treatments with psychedelics or antidepressants are *augmented* by psychotherapy and/or significant environmental changes.^154^

## 6 Conclusions

### 6.1 Comparison of Cholesterol with Select Leads from HTS

From the previous Section , TrkB PAMs based on steroid, or steroid-like, structures are good candidates for binding the cholesterol binding site of TrkB. Based on their structures alone, this seems highly likely for dehydroepiandrosterone, BNN-20, ENT-A010, and others,^155–157^ quite likely for deoxygedunin, and a possibility for 7,8-dihydroflavone, its derivatives, and gambogic amide (Figure 6). For most of these compounds, we think it would be surprising if they did not bind the cholesterol binding site to some degree. This does not exclude the possibility they may also bind the extracellular domain, but they seem particularly suited to interact with extended hydrophobic protein pockets, which are rarely accessible in polar media.

**Figure 6:**
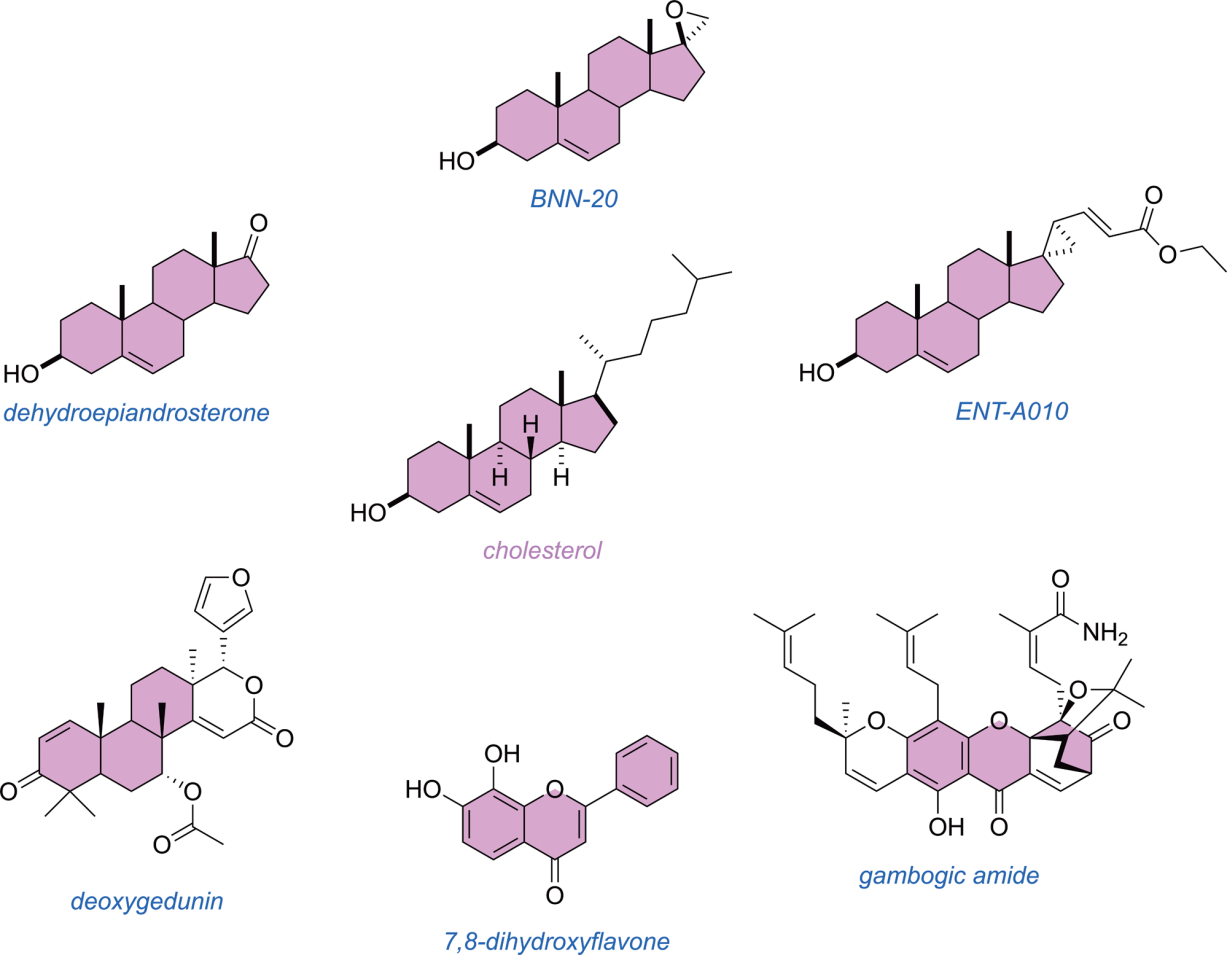
Structural similarities between cholesterol and putative TrkB agonists.

### 6.2 Comparison of Psychedelic/Antidepressant TrkB PAMs with Select Leads from HTS

Recall from the section on PAMs that LSD and PSI strongly bind to a site close but distinct to the cholesterol binding site, and antidepressants fluoxetine, imipramine, and ketamine bind less strongly to an overlapping site. Figure 7 highlights structural similarities between the psychedelics and antidepressants and the putative TrkB agonists DMAQ-B1, NAS, amitriptyline, and deprenyl. Deprenyl was known as a clinically used antidepressant before it was identified in high-throughput screens as a putative TrkB agonist.

**Figure 7:**
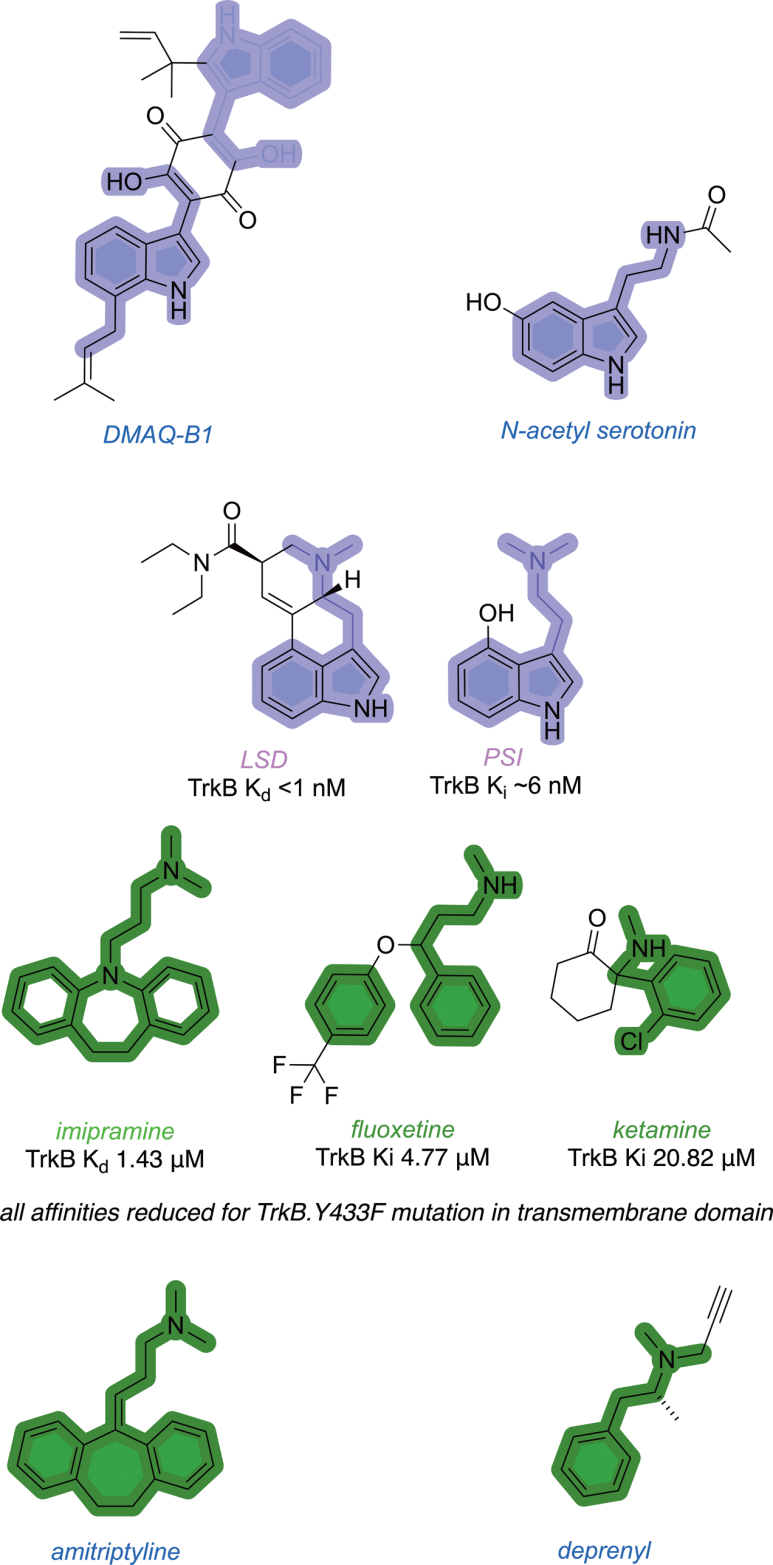
Structural similarities between psychedelics and antidepressants that bind the TrkB transmembrane domain and putative TrkB agonists.

Just as for the steroid derivatives in Figure 6, structural similarities like these do not prove that the putative TrkB agonists bind its transmembrane domain and by inference are not true agonists. Neither do they establish if any of these compounds bind both the extracellular and transmembrane TrkB domains. However, they do introduce uncertainties about whether these putative TrkB agonists act in that way or function primarily as PAMs.

### 6.3 Other Putative Trk Agonists in This Review

Other notable putative Trk agonists mentioned do not overlay neatly on steroidal backbones, psychedelics, or antidepressants but are nevertheless small amphipathic, predominantly hydrophobic compounds, which might be expected to favor binding to a hydrophobic transmembrane region, over more polar facing extracellular environments. These mostly include various LM and PTX structures originating from Longo, notably LM11A-31, which is now in clinical trials for Alzheimer’s disease.^158–162^ Referring back to Figure 3, most structural types discussed in this review are putative TrkB agonists. It could be more than a coincidence that only TrkB, not A and C, has a cholesterol binding site.

As an aside, some TrkA ligands reported to be PAMs;^163^ how could this be explained given that TrkA does not have a cholesterol binding region? In several ways. Some PAMs could bind Trk *extracellular* regions. Alternatively, they could bind lipophilic transmembrane regions that are not cholesterol binding sites (*cf* psychedelics bind sites distinct from the cholesterol binding site in TrkB, so perhaps small hydrophobic molecules could bind TrkA and C TMD regions, even though there is no CRAC or CARC domain). In the discussion above, we describe experiments featuring chimeras of the form TrkB·TrkA.TMD, where TrkB PAMs did not induce the same results with those receptors as with wild type TrkB. However, as far as we are aware, “inverted” chimeras of the type TrkX·TrkB.TMD have not been reported in the studies we reviewed. Recall that neurotrophins are not specific for one Trk binding site (Figure 1a), so it seems likely NGF, NT-3, or NT-4 could synergize with a TrkB PAM to cause signaling at TrkB.

We do not imply PAMs, discovered accidentally or not, cannot be as effective as or more effective than Trk agonists. To the contrary, a recent review highlights the considerable potential of Trk PAMs as therapeutics for Alzheimer’s disease.^159,164^ Further, some of the compounds shown in Figure 3 were not emphatically described as Trk agonists. Instead, our points are these. Many of the putative Trk agonists were screened because they had some combination of properties, like small molecular mass, blood brain barrier permeability, amphipathic/predominantly hydrophobic properties, and known impacts on the brain. For instance, compound collections assembled for HTS would deliberately include drugs (like deprenyl^89^), some of which are known to act in the brain (e.g., amitriptyline^53^), or endogenous neurochemical metabolites (e.g., NAS^54^). Another major component of the libraries was natural products, which might be expected to show a range of activities (e.g., flavonoids like 7,8-DHF,^56^ alkaloid-like compounds like gambogic amide,^52^ quinones as in DMAQ-B1,^75^ and ones resembling steroids such as DHEA^98^) because of nonspecific binding, or suppression of reactive oxygen species (particularly for flavonoids). Most were screened with the intention of binding to Trk extracellular domains and inducing phosphorylation. Indeed, primary assays for pTrk were routinely featured. It now seems likely that many do not act in that way, based on recent discoveries by Castrén and others.

It is unsurprising certain chemotypes in the libraries used for HTS show activities in assays featuring neuronal cells or neurological conditions because these libraries were biased to include neuroactive substances in the first place. Further, some were known to be nonspecific binders that might be expected to display polypharmacology. Polypharmacology is double-edged. It can concurrently lead to a range of therapeutic modalities (e.g., aspirin) but also ambiguous modes of action and extensive side-effects. Molecules of this type are poor probe candidates for cell signaling or for in vivo models for disease states in which the mode of action is important, because they are not selective. Most of the HTS leads discussed in this review are not selective enough to be probes due to the above issues. They may not be optimal drug candidates, for other reasons. Patent protection, and ultimately drug approval, of well-known psychoactive drugs with moderate in vivo activities acting via more than one mechanism is not easy. This is particularly so if the compounds have significant cytotoxicity (e.g., DMAQ-B1) or undesirable side effects (e.g., amitriptyline).

### 6.4 Leads Discovered by Virtual Computational Screening

Aspects of structure-based design was used for some of the putative Trk agonists. Informative structure-based design depends on (i) extent and quality of protein ligand, receptor, and ligand·receptor structural data; (ii) soft and hardware resources; and (iii) degree of simulation rigor. Since Trk·neurotrophin structural data are mostly lacking for the regions involving hot-loops, especially in and around the transmembrane region where neurotrophins bind, this is a major limitation.

Docking studies usually search for small molecules with pharmacophores simulated to fit compactly and with high affinity into target receptor cavities. However, small-molecule pharmacophores do not closely resemble endogenous protein ligands. Further, involvement of lipophilic scaffolds prone to nonspecific protein binding are likely to give polypharmacologies, like many of the HTS leads. For instance, LM11A-24 and LM11A-31 are structurally related to purine bases/caffeine and iso-leucine. LM22A-4, LM22B-10, and PTX-BD10-2 have hydrophobic cores and relatively simple structures that medicinal chemists might anticipate would bind more than one target. Some of the leads discovered via computational screens impact Trk receptors based on the evidence reviewed here, but overall, there may still be concerns about target specificity. Modeling data alone are not strong enough to implicate binding to extracellular domains over hydrophobic regions in transmembrane ones. It would be interesting to see if and how these molecules bind the TMDs in molecular dynamics experiments of the type used by Castrén and co-workers featuring Trk TMDs (e.g., 5a): a kind of virtual counter screen.

### 6.5 Cell Used in Assays to Detect Trk Agonism

Disagreement about whether several of the putative Trk agonists act via this mechanism was perhaps a clue that an alternative mode of action, such as PAMs, might be operative. Castrén’s work highlights reasons inconsistencies might arise between laboratories. First, it shows cholesterol levels in membranes are highest at the synapses and govern the amount of active, functional crossed TrkB dimers displayed. It is not known how removing primary cells from mice and rats impacts cholesterol levels in the membrane. Neither do we know how cholesterol levels vary for stable transfectant cells after repeated culture or with the parent cell line they were cultured into. Second, primary cells may secrete relatively low levels of neurotrophins. When this occurs, a PAM binding a Trk transmembrane region may appear to directly generate phosphorylation, when it might instead be synergizing with the endogenous neurotrophin.

Tests on cells transfected with Trk receptors are attractive insofar as Trk or p75 receptors introduced can be expressed in parent cell lines that do not express them otherwise. Using transfectants is convenient because culture tends to be faster and more reliable for tumorigenic cell lines than for primary neuronal ones. This makes the experiments robust and less time-consuming. However, Trk activation in cells transfected into readily available cell types may proceed in ways which do not resemble those in human brain. For instance, kidney cells like HEK293 and murine embryonic fibroblasts NIH3T3 have membrane compositions, cell morphologies, and internal biochemistries that neuronal cells do not share. Moreover, ratios of densities and expression levels of the featured receptors (e.g., TrkB-p75 combinations) can impact cell signaling.

Primary neuronal cells are harder to acquire than tumorigenic lines commonly used for transfectants and cannot be obtained routinely from humans. They probably provide more authentic environments of Trk and p75 receptors than transfectants, but using them involves significant wild-card issues. These lines rarely express TrkA, B, C, or p75 exclusively, and they also bear many other surface neuroreceptors that can have variable responses to nonendogenous small molecules. This is a significant concern for simple hydrophobic molecules that lack Trk binding specificities. If the cells used are extracted from parts of the brain with tendencies to produce one of the featured receptors selectively, then they may not be relevant to other regions of the neuroanatomy these compounds may activate in vivo. Further, the homogeneity of these cells must be impacted by the degree of precision by which these cells are extracted. Conditions under which the cells are maintained will impact their cell surface receptor expression profiles, lipid and glycoprotein compositions, and excretion of neurotransmitters. Excreted neurotransmitters could be small molecules like glutamate and GABA and cytokines like glial cell-line-derived neurotrophic factor (GDNF). In the brain, characteristics of neurons are impacted by surrounding cell types like glial and astrocytes, environments that cannot be accurately replicated in cell culture.

Despite the many and serious limitations outlined above, combinations of induced cell survival in the transfectant and in primarily neural cells are possibly the most informative assay sets available. However, at best, they are crude indicators of the brain environment, and they are vulnerable to many variables that could introduce batch-to-batch and lab-to-lab discrepancies.

### 6.6 Assays to Detect Intracellular Phosphorylation

All ambiguities and relevancy issues implicit in cell line selection (as outlined above) apply to blotting assays featuring blotting for phosphorylation and signaling events in their lysates. Difficulties detecting phosphorylation overlay and amplify these issues.

Selectivity and sensitivity of Abs to detect pTrk may be suboptimal because intracellular Trk receptor regions could vary their conformations and compositions as they interact with other membrane biomolecules, and their autophosphorylation patterns consequently may involve different combinations of amino acids. Further, anti-pTrk Abs are not validated by a wide customer base. Blots featuring them are rarely clear, and some are not shown in any publications. Some researchers compensate for poor gel qualities on crude lysates by immunoprecipitation with Trk Abs prior to blotting for pTrk or pTyr. Immunoprecipitation requires this additional step, slowing throughput, making it more arduous to validate the assay overall but significantly increasing signal-to-noise.

ELFI and ELISA variants outlined in this review circumvent limitations of pTrk Abs, and they have better throughput, making statistical validation less arduous. These assays test for downstream targets like pERK, pAKT, and pMAPK using robustly validated Abs. However, they are not used throughout all of the studies involved. Related to this, we have already discussed how negative findings were reported by more than one group for the most widely accepted TrkB leads (e.g., for many in Figure 3). Further, there is a consensus among those who question some reported Trk agonists. The three main dissenting groups (Bard, Sames, Matłoka) searched for phosphorylation using combinations of assays outlined above and between them only found that DMAQ-B1 induces Trk phosphorylation.

Phosphorylation kinetics is another complicating issue. Neurotrophins typically induce maximum levels of pTrk after 30–60 min incubation in ELISA,^70^ whereas small molecules diffuse much faster into receptor regions and bind with faster on and off rates. Small molecules inducing any detectable pTrk typically do so maximally after 3–20 min in these assays.^70^ Thus, observation of phosphorylation for novel small molecules might maximize after brief, unpredictable times, which may be too short to observe under routine conditions. Searching to test *if* an event occurs and simultaneously *when* it happens is an unenviable task because of the lack of confidence in negative data. “Optimization” of phosphorylation kinetics is a misnomer if there is uncertainty about whether there is any; “random sampling” of incubation times may be a more appropriate description.

Longo’s team, key discoverers of putative small-molecule Trk agonist leads, has proposed many of these leads act as partial agonists.^165,166^ A partial agonist is a compound that activates the receptor in question to a lesser degree than native ligand.^166^ This means that when a partial agonist and native ligand are both competing for the same binding site, receptor activation (measured at Trk phosphorylation in this case) is observed to decrease relative to the maximum response of the Trk receptor to NTs. We concur with this rationale, and in unpublished work, we similarly have observed these partial agonist-like characteristics of phosphorylation for small molecules at high concentrations of NT that *enhanced* cell survival in the presence of suboptimal NT concentrations. It is entirely possible that small molecules may antagonize NTF, or give undetectable Trk phosphorylation in isolation, *and conversely* positive cell growth or survival outcomes. However, as we have discussed, there is also the possibility that these compounds act partly or primarily as PAMs binding the TMDs.

When suboptimal amounts of NT are added in assays for Trk agonism by small molecules (cell survival or phosphorylation), a positive outcome would be synergy, but this would *not* be observed in the situation discussed above. However, at face value, true agonists (that act in the absence of NTs) tested in the absence of NTs should give pTrk for cells that do not produce exogenous NTs, but for neuronal cells, positive pTrk is less likely to be observed.

Overall, we conclude observation of pTyr by blotting and related methods may not be possible due to any of the following factors alone or in combination: (i) necessarily inappropriate cells and culture conditions; (ii) inadequate selectivities and/or batch-to-batch fidelities of Trk Abs: (iii) limitations of throughput making validation difficult with available resources; (iv) inconveniently fast and unpredictable phosphorylation kinetics; (v) competition of less potent small molecules with relatively potent NTs added or excreted in small amounts; and (vi) if the compounds are PAMs not any type of agonist. Further, small-molecule agonists may diffuse in and out of Trk receptors with relatively high frequencies, inducing cumulative cellular effects in a static incubation experiment, yet have different modes of action in vivo.

### 6.7 Assays Featuring Engineered Cells with Reporter Systems Linked to Trk Activation

Cells of this kind include those for the TrkB-CHO CellSensor assays utilized by Sames and Bard. Neither of their groups successfully confirmed activities for any of the key small molecule leads (Figure 3) via these approaches.^69,70^ We did not note other groups using these approaches to successfully detect small-molecule TrkB modulators either.

Assays of this kind have all the issues surrounding suitability of cell lines and culture discussed above and burdens associated with activation kinetics. Neurotrophins tend to give maximal signal through Trk or downstream effectors around 30–60 min via ELISA, ELFI, or blotting, and small-molecule Trk modulators do so faster.^56,70^ Rapid transient signals induced by small-molecule activation may be below detection limits of such assays, or at least the cellular factors contributing to the readout may be more complex than relying on a more direct measure of phosphorylation. Regardless, most (with the exception of DMAQ-B1) of the small molecule leads that did not induce a signal in this assay were similarly negative in the ELFI and ELISA assays at short time points (<15 min) as well.^69,70^

DiscoverX PathHunter is different insofar as it relies on noncovalent recombination of two complementary β-galactosidase fragments; hence, positive responses occur on much shorter time scales. While the TrkB in this assay has been validated as functional^70^ (BDNF induces phosphorylation of Erk in ELFI), hits found through this assay were not able to be confirmed via ELISA or ELFI. Further, the PathHunter assay appears to be inconsistent in reproducing activity measured by other methods.^69,70^

Finally, these assays detect agonistic activity, but they would not detect PAMs unless NTF is also administered. This could be the reason this assay did not work for many putative Trk agonists, which are actually PAMs.

Overall, we conclude that the chances of false positives or negatives for small-molecule agonists are high in engineered cells expressing Trk coupled to reporter systems, even if NTs or anti-Trk Abs give positive outcomes in control experiments with NTFs. False positives should become evident in secondary assays. Negatives are unfortunate, however, because short transient activation of the receptor tyrosine kinase system in cellular assays could be associated with durable responses in vivo. Neurotrophins are short-lived in vivo, but more durable small molecules can have potential for repeated activation of Trk receptors.

### 6.8 Binding Assays

Problems with binding assays for cell surface receptors are widely appreciated. Isolation of Trk receptors has several issues, beginning with the cells used. Isolated Trk receptors are vulnerable to proteolytic cleavage, and their conformations and glycosylation states may change in alien environments (e.g., detergent additives). Supported extracellular Trk domains are therefore crude surrogates for Trk receptors displayed on neuronal cells in vivo. Further, direct binding assays based on mass increases on binding supported receptors (e.g., surface plasmon resonance or biolayer interferometry) are inaccurate and insensitive for small molecular mass substrates. Sensitivities in calorimetry experiments are dependent upon enthalpies liberated during the binding event and limited by the quantities of proteins available.

Competitive binding experiments using neurotrophins (or anti-Trk Abs) are flawed if binding regions of these exogenous protein ligands are changed by the Trk receptor isolation procedure or for small molecules that bind Trk in dissimilar ways. Throughout, if the biologically significant event is binding of a small molecule to a Trk·p75 complex in an adaptable cell membrane, then that may not be reproduced with isolated cell surface receptors.

### 6.9 In Vivo Assays

Implantation of human tumors into immune-compromised mice to identify therapeutics that regress cancer pathology is highly relevant to humans. On the other hand, rodent models are necessarily less realistic representations of human neurological diseases due to the complexities of neuropathology formation and progression. Consequently, translation from preclinical candidates validated in animal models into clinically viable pharmaceuticals is particularly difficult for any maladies related to the brain. Nonetheless, genes encoding Trk receptors and neurotrophins are highly conserved across all mammals, including between rodents and primates, and rodent models of neurological diseases are currently our best method to identify suitable drug candidates before clinical testing is initiated. Nevertheless, in transformation from preclinical candidates validated in animal models into human trials, it is still possible small molecules act as Trk modulators in mice and predominantly on some different targets in humans.

### 6.10 Small-Molecule Trk Modulators: Where Do We Go from Here?

Our thoughts are summarized in Figure 8. All of the early stage assays outlined above have unavoidable flaws and limitations, and the significance of these may be difficult or impossible to assess. Every assay using cells transfected with Trks or primary neuronal cells ex vivo has issues. There do not appear to be more reliable options.

**Figure 8:**
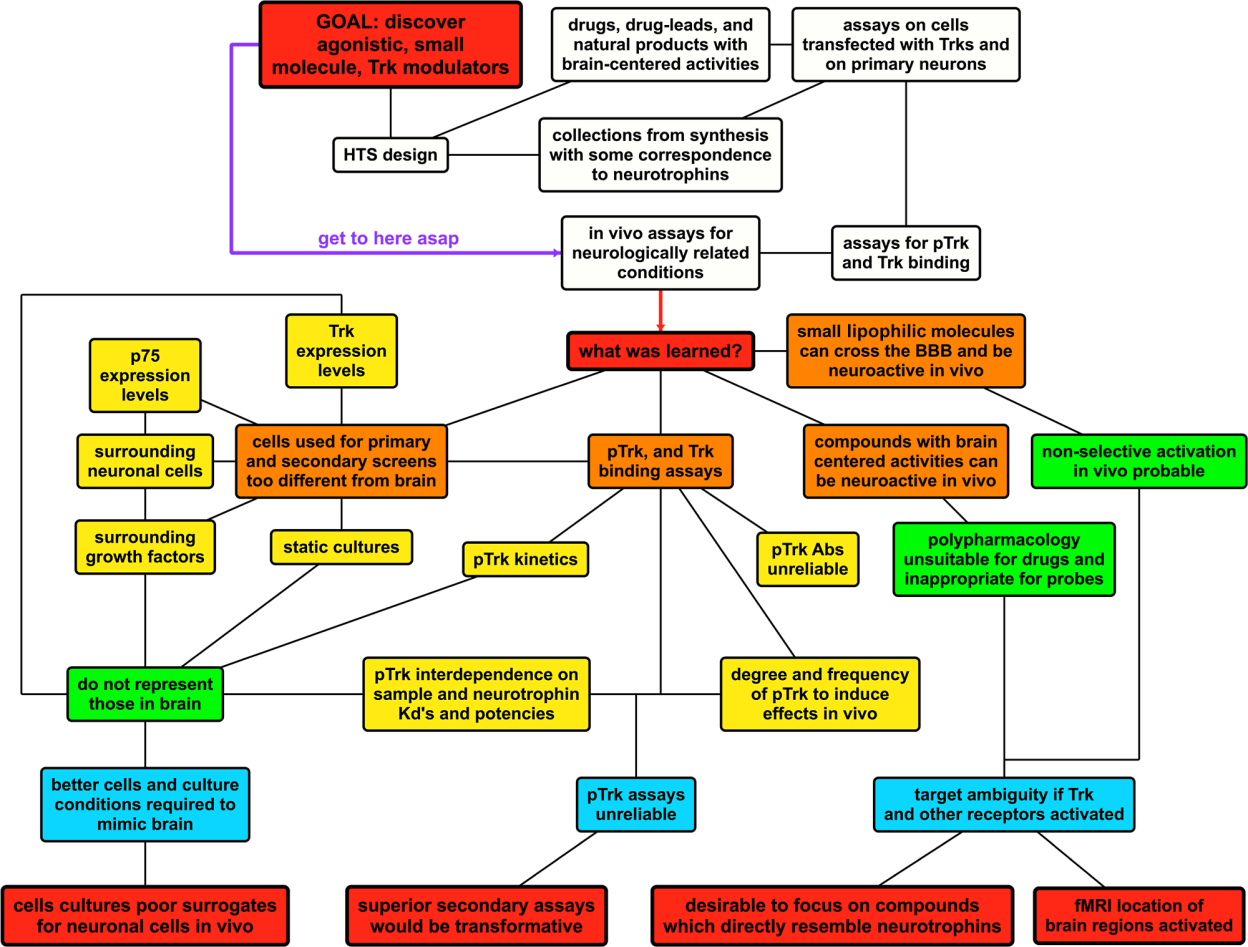
Lessons from studies on small-molecule Trk modulator candidates.

It follows that selection of putative hits for in vivo studies must involve higher false negative and positive rates, and misinterpretations may result. Gaps between cellular and in vivo studies in this area are huge. This is extremely important because in vivo tests for activities associated with the brain are significantly more time-consuming and expensive than primary screens. Thus, researchers are forced to select small molecules for advanced assays without being confident they have optimal leads or to predict outcomes in vivo, even if only a Trk, or Trk combination, is activated. Establishing pathways of action of putative Trk modulators in vivo is even more difficult. How neuronal stimulation occurs is difficult to determine under the best circumstances and may be impossible if these leads have polypharmacology.

We have not seen any approaches to answer the hard question: how to bridge the gap between cellular and in vivo disease models for small-molecule Trk modulators and cannot suggest obvious strategies to do so. Such assays might not be possible with the tools currently available. Until better assays are available, we advocate using these criteria for choosing chemotypes screened in HTS from the onset:

1 Compounds biased to nonspecific binding and polypharmacology should be excluded, particularly hydrophobic ones with little functionality.
2 Chemotypes based on synthetic structures (often pharmaceuticals) with known brain-centered activities involving other targets should be excluded if there is no prospect the neuronal target combinations in an in vivo study can be identified.
3 Pharmaceuticals with known detrimental side-effects should be excluded from studies aimed at producing safe drugs.
4 To identify molecules with true agonistic activity, emphasis might be given to chemotype designs that relate more closely to neurotrophins structures than the small molecules described in this review.

Criteria 1–3 are negative exclusion principles, and the only positive one, 4, is perhaps contentious, but justifications in favor of 4 are as follows. Chemotypes with clear structural correspondences to neurotrophin structures would include peptide fragments likely featuring natural amino acid side chains since these are predominant pharmacophores in natural NT·Trk complexes. Modified designs might also include nongenetically encoded amino acids, or analogues, bearing side-chains with close physiochemical similarities to the ones in NT hotspots for binding Trk receptors. In our view, it is opportune to mimic NTs. Chemotypes with closer structural parallels will tend to be more selective for their intended Trk targets and less likely to strongly bind and activate elsewhere. *Nature* uses this approach in evolution: combining just 19 or 20 side chain pharmacophores to give high selectivities and efficacies. Diversity, and therefore selectivity, results from the huge numbers of sequences and conformational biases that can be evolved from these building blocks to induce neurological effects. This is hard to mimic with nonpeptidic small molecules.

Criteria 4 above include cyclic-peptides and peptidomimetics. Chemotypes of this kind have been tested, including some from our limited and modest research in this area.^175^ Peptides, cyclic-peptides, and peptidomimetics resembling NT fragments have been reviewed previously.^46,50,167–171,174^ Compared with small molecules, fewer in vivo studies have been performed, and clinical trials featuring these chemotypes are rare.

Looking ahead, perhaps small-molecule fMRI studies in response to treatments involving safe, putative Trk modulators would be informative. fMRI comparisons could be made between animal subjects and clinical trial participants, or afflicted and healthy humans, and treatment with neurotrophins and with test samples would give interesting comparisons.

Readers who disagree with criteria 4 might accept the following overall conclusion. Selection of compounds for testing as potential agonistic modulators is critical. It is unproductive to include nonselective binders when searching for probes or toxic substances when pursuing clinical targets. Many commercial libraries are similar insofar as they feature drugs, drug candidates, and natural products for repurposing, or they comprise molecules easily accessed by synthesis. Computationally guided design of molecules is important, and these analyses studies should not be limited to commercially available compounds or ones easily prepared via simple chemistries used extensively in library construction; better chemotype selection increases the chance of finding hits from relatively small libraries. This review illustrates how virtual library compositions to select compounds for experimental screening are critical to bias hits to selectivity and away from toxicity. Further, better assays to differentiate PAMs from true Trk agonists and to gauge effects on neuroplasticity should be developed.

Trk and neurotrophins are heavily implicated in the regulation of synaptic plasticity and associated diseases.^172^ Alterations in synaptic plasticity has long been linked to psychiatric disorders, including PTSD, major depressive disorder (MDD), and substance abuse, among others.^173^ Therefore, it can be beneficial to identify Trk-modulating compounds that promote synaptic plasticity to discover efficacious compounds toward psychiatric disorders. However, there is a lack of screening methods to directly identify modulators of synaptic plasticity in a high-throughput manner. This may be due to the tedious nature of assessing a compound’s potential for promoting synaptic plasticity. There are 2 routine methods in determining the influence of a biological agent on synaptic plasticity. The first is to conduct electrophysiology on cultured neurons, measuring postsynaptic potentials, which would have to be conducted one compound at a time, taking over an hour per compound just to conduct the experiment. The second is conducting histological analysis of the brains of treated animals. This can be more convoluted, requiring optimization of the dosing regimen through pharmacokinetic analysis and the laborious process of analyzing tissue for changes in synaptic plasticity, which includes high-magnification imaging and manual quantification of synaptic terminals.

It would be unfortunate if grant reviewers solidify biases against further investigations of Trk because so much effort has been expended on the HTS of *non*-*peptidic* small molecules. Improved structural data, possibly from high-resolution electron microscopy, and advances in our understanding of cell signaling processes might drastically accelerate effective development of Trk probes using these chemotypes. Related to this, we are particularly impressed by the work of Castrén and co-workers. That work profoundly impacts studies of antidepressants and use for psychedelics for depression and posttraumatic stress disorder.^153^ They also impact the controversies surrounding illegal microdosing of some psychedelics to improve creativity via improved neuroplasticity. Castrén’s work is fundamental research, not featuring HTS and not intended to develop new Trk ligands. Nevertheless, it is poised to have a profound impact on this area.

## Abbreviations

BBB: blood brain barrier
BDNF: brain derived neurotrophic factor
BPD: bipolar disorder
CRAC: cholesterol recognition/interaction amino acid consensus
CARC: inverted CRAC
DHEA: dehydroepiandrosterone
7,8-DHF: 7,8-dihydroxyflavone
DMAQ: demethylasterriquinone
EGFR: epidermal growth factor
ELFI: enzyme linked fixed cell immunoassays
EPOR: erythropoietin receptor
FRET: Forster resonance energy transfer
FYN: raft-restricted kinase
GF: growth factor
GFP: green fluorescent protein
HUVEC: human umbilical vein endothelial cells
IR: insulin receptor
LSD: lysergic acid diamide
LTP: long-term potentiation
MAO-B: monoamine oxidase-B
MCAO: middle cerebral artery occlusion
MDHB: methyl 3,4 dihydroxybenzoate
NAS: *N*-acetyl serotonin
NGF: nerve growth factor
NT: neurotrophin
NT-3: neurotrophin-3
NT-4: neurotrophin-4
PAM: positive allosteric modulators
PLCγ1: phospholipase C gamma 1
PPI: protein–protein interaction
PSI: psilocin
Trk: tropomyosin receptor kinase
